# Cell competition driven by secreted ligands: Modeling liver metastasis of colorectal cancer

**DOI:** 10.1073/pnas.2531078123

**Published:** 2026-08-27

**Authors:** Hossein Nemati, Saskia Jacoba Elisabeth Suijkerbuijk, Joost de Graaf

**Affiliations:** ^a^ Institute for Theoretical Physics, Department of Physics, Faculty of Science, Utrecht University, Princetonplein 5, Utrecht 3584 CC, The Netherlands; ^b^ Division of Developmental Biology, Institute of Biodynamics and Biocomplexity, Department of Biology, Faculty of Science, Utrecht University, Padualaan 8, Utrecht 3584 CH, The Netherlands

**Keywords:** cell competition, cell cycle, consumer–resource modeling, physical modeling, mean-field model

## Abstract

Cell competition in multicellular organisms has been shown to play a critical role during the development of organisms, cancer progression, and in the establishment and maintenance of tissue homeostasis. Various mechanisms of cell competition have been identified, including active elimination via mechanical forces or induced apoptosis, as well as competition for nutrients and other beneficial factors. A recent experiment demonstrated hallmarks of cell competition, associated with cell cycle dynamics, between liver progenitor cells and colorectal cancer cells [A. Krotenberg Garcia et al., *iScience* **27**, 109718 (2024)]. However, a mechanistic explanation for this form of competition remains lacking. Here, we present a mean-field model of competition for signaling ligands, coupled with cell cycle dynamics, to provide such an understanding. Our model captures the salient features of the experiment, including population dynamics and cell cycle variations. We demonstrate that secretion of a beneficial factor by cells, coupled with the enhanced uptake efficiency of cancer cells, suffices to reproduce the experimental outcome. Our model, reminiscent of competition for secreted growth factors, provides insight into the minimal level of complexity required to achieve the observed competitive outcome as well as its link to cell cycle dynamics. It can also serve as a general framework for studying biological populations with growth-stage-dependent competition over consumer-produced products.

## Significance

Cell competition is relevant for a range of biological processes, from the elimination of malignant cells in tissues to cancer progression. It can arise through various mechanisms, including signaling pathways or ecological competition for nutrients and secreted factors. However, in many cases, the underlying mechanisms remain poorly understood. Here, we present a model of cell competition mediated by secreted factors in cell populations. Inspired by the competition observed experimentally between colorectal cancer and wild-type liver cells in a previous study, the model successfully reproduces the experimental findings. Beyond cell biology, this framework can be extended to any society of living agents that simultaneously produce and consume shared resources in a competitive manner.

Cell competition is an evolutionary process whereby cells with high survival and proliferative capacities, reflecting higher fitness, are preferentially selected over less-fit cells (1, 2). This process can occur through direct cell–cell interactions, secretion of biochemical factors, or mechanical forces (2–5), which in some cases can even result in the elimination of neighboring cells (5–9). Cells may also compete for limited shared resources in an ecological manner (10–15), although this is not traditionally categorized in cell competition in the literature of cell biology. The concept of cell competition was first introduced in 1974 by Morata and Ripoll, who observed disappearance of mutant cells when surrounded by wild types in the *Drosophila* wing disc (16). Since that time, the mechanisms of competition have been clarified through a series of studies on interactions between different cell types in *Drosophila* (12, 17–22) and more recently in mice (6, 8, 23–28). A historical review of cell competition studies was recently performed by Morata, himself (29).

Cell competition processes are generally classified into two categories: active and passive competition. In active competition, one cell type interferes with the behavior of another through mechanisms like induction of apoptosis (6, 12, 17, 30–33), differentiation (7, 28), or cell-cycle modifications (6, 23). In contrast, passive cell competition is characterized by the absence of interference between cell types, although a selective advantage may still arise for one type over the other (1, 2) through, for example, specific mutations (34–36) or position-dependent advantages (37).

Cell competition is a double-edged sword. On the one hand, competition can serve as a quality control mechanism for the integrity of tissues in development or in homeostasis (1, 38). On the other hand, certain types of cancer abuse competition to increase their proliferation capacity (2, 4). For a deeper biological insight into cell competition mechanisms, the reader is referred to recent comprehensive reviews in refs. 1–5.

Numerous scientific studies have explored the modeling of cell competition. While a comprehensive review of all modeling approaches is still lacking, partial overviews can be found in refs. 39 and 40. Here, we briefly summarize the three major lines of modeling, in order to contextualize our work. The first category includes models based on ordinary differential equations (ODEs), which describe the dynamics of different populations and their essential (shared) resources using differential equations. The populations can be interpreted as cell types, which interact indirectly via resource competition, or directly, as is the case for predator–prey dynamics. Prominent examples of such models include MacArthur’s model (41, 42); Tilman’s model (43); and the Lotka–Volterra model, as well as its generalizations (44–46). For instance, the Lotka–Volterra model has been used to model competition among breast-cancer cell lines (11). These models can also connect the cell cycle and com petition for shared resources (47–50).

The second category of quantitative models originates from the fields of evolutionary dynamics and population genetics. These models are typically based on stochastic processes that govern population dynamics through discrete time steps (51, 52). Prominent examples include the Moran (53) and Wright–Fisher (54, 55) models. Buder and colleagues have used a simple Moran model to study tumor progression for a range of tissues (56). These frameworks can also be extended to cover structured populations using graphs, which opens the field evolutionary graph theory (57). Game theory is also used in these models to describe different types of competitive interactions between individuals (58, 59). However, a limitation in this category is that many detailed biological properties are encoded in aggregated quantities such as fitness and game payoff, that do not distinguish between individuals of the same type.

The third category of quantitative models are cell-based models, also known as agent-based models. These models consider cells as physical entities and describe their interactions using equations of motion based on potentials or forces. Examples of these models are particle-based methods (60–65), the vertex model (66–70), Voronoi models (71–73), cellular Potts (74–79), and multiphase-field models (80–82). For the interested reader, these modeling approaches are comprehensively reviewed in ref. 83. These frameworks have been applied to study, for example, competition for resources (84), and competitive interactions arising from the mechanical properties of cells (85–87), cell motility (88), cell density effects (89), and cell cycle dynamics (65, 90).

In a recent experimental study by Krotenberg Garcia and colleagues (23), cell competition was observed between liver progenitor cells and colorectal cancer cells, a finding relevant given that liver is the most common site of colorectal cancer metastasis (91–93). The observed competition manifested as asymmetric variations in proliferation rates of the two types compared to their pure conditions, as well as alteration in the distribution of liver cells across cell cycle phases (23). In the experiments, nutrients were abundant in the medium. This condition was imposed, such that Krotenberg Garcia et al. (23) could be certain that the bottleneck for competition is cell–cell interaction rather than passive competition for nutrients. Despite the experimental evidence of competition, the specific type of signaling or cell–cell interactions responsible for these observations remains unknown and is still under investigation. In this work, inspired by the experimental observations (23) and motivated by the pursuit of a minimally complex model, we propose a mean-field framework that captures the experimental results by considering competition for secreted signaling ligands. Modeling studies have explored cell competition and its interplay with the cell cycle in terms of active competition (89, 90), mechanical competition (65, 85), and competition for externally provided resources (48–50). However, scenarios involving competition for secreted ligands and their impact on cell cycle dynamics remain poorly characterized. Our model provides a plausible mechanism linking this form of competition to cell cycle regulation within a minimal, unified framework. From the broader perspective of consumer-resource modeling, this model incorporates growth-stage-dependent competitive behavior and the dynamics of consumer-produced resources. Our work can also be extended to ecological scenarios where similar competitive behavior is relevant.

## The Experimental Observations.

Krotenberg Garcia and colleagues (23) have recently studied cell competition between liver cells (either progenitor cells or hepatocytes) and colorectal cancer cells, in the context of colorectal cancer metastasis to the liver, using mixed murine organoids and microtissues. Here, we briefly review the results most relevant to our study, that is competition between liver progenitor cells and colorectal cancer cells. We refer the reader to refs. 23, 94, and 95 for details of the experiment.

**Fig. 1.**
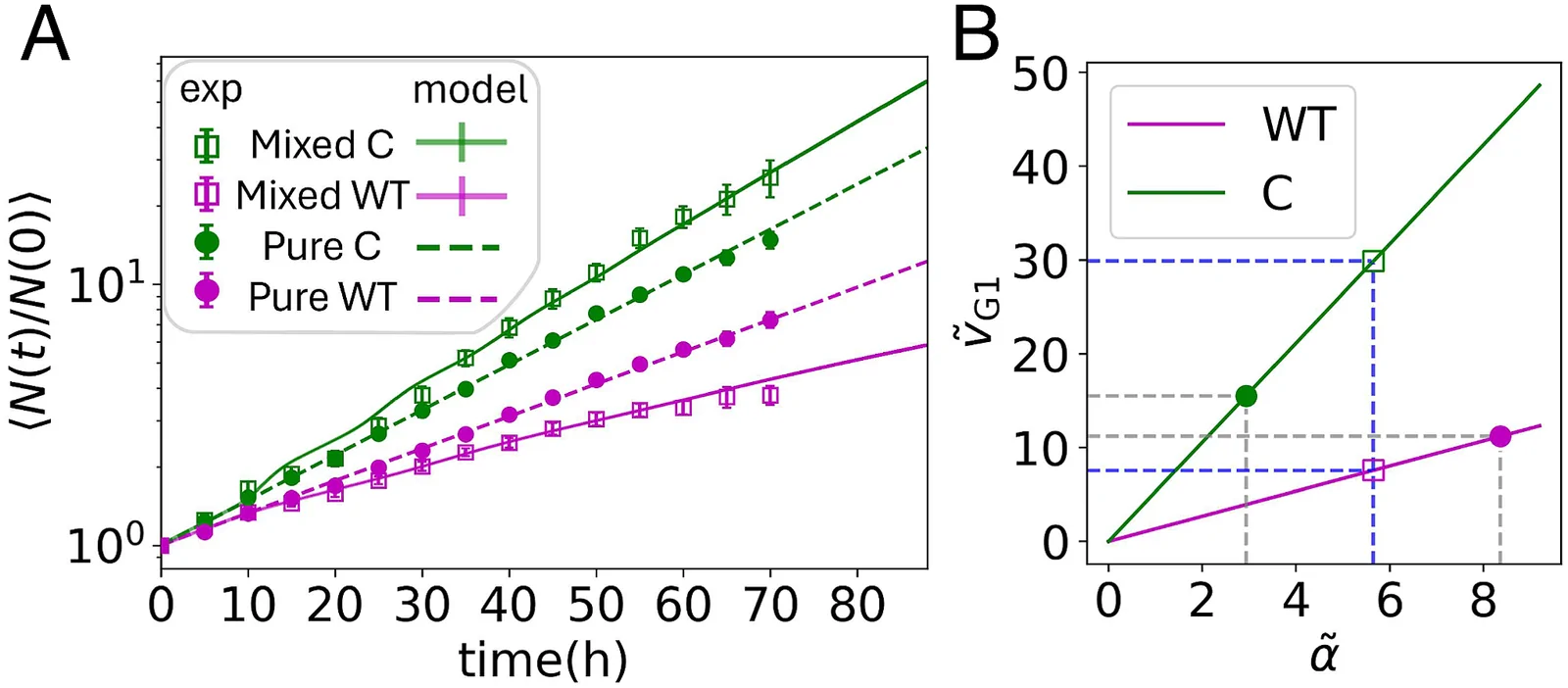
Competition in terms of population growth. (*A*) Normalized population of wild-type and cancer cells are plotted. The curves are from the model, while the data points are the experimental data. Labels “Pure WT” and “Mixed WT” indicate the population of the wild-type cells in the pure and mixed organoids, respectively. The labels “Pure C” and “Mixed C” have similar meanings for cancer cells. Uncertainties indicate SE of the mean (SEM). (*B*) Progression speed in G1 phase, ${\tilde{v}}_{G1}$, as a function of growth factor concentration, $\tilde{\alpha }$, for wild-type and cancer cells. The solid circular markers show the equilibrium points in the pure conditions. The open square markers show the initial values of ${\tilde{v}}_{G1}$ for wild-type and cancer cells in an example mixed organoid with a 50 : 50 composition. The gray and blue dashed lines serve to guide the eye. The full definitions of the quantities $\tilde{\alpha }$ and ${\tilde{v}}_{\text{G1}}$ are provided in *Model Formulation*.

The most important result reported by Krotenberg Garcia et al. (23) is the population growth of the two cell types. The populations of wild-type and cancer cells in both pure and mixed organoids were studied. In pure conditions, the populations of both cell types grew exponentially, with a higher rate for cancer cells. By performing a weighted least-square fitting on the experimental data to curves $N\left(t\right)=N\left(0\right)\;e^{\beta t}$, we found $\beta_{W}=0.0284\pm 0.002\;{\text{h}}^{-1}$ and $\beta_{C}=0.0398\pm 0.002\;{\text{h}}^{-1}$ for pure wild-type and cancer populations, respectively. Here, β represents the (exponential) growth rate, i.e., fractional population increase per unit time. It is related to the population doubling time, $\tau_{d}$, through $\beta =\;\ln2/\tau_{d}$. However, in mixed organoids, the growth of both cell types deviates from these exponential curves. Specifically, cancer cells in mixed organoids proliferate faster than in pure conditions, while the proliferation rate of wild-type cells decreases, as shown in Fig. 1*A*. This clearly indicates a form of cell competition between wild-type and cancer cells in mixed organoids, mediated by interactions between the two cell types. However, as indicated previously, the precise mechanism underlying these interactions remains unknown.

The mixed organoids had a range of starting ratios, which revealed that the deviation from pure conditions in one type was exacerbated by a larger proportion of the other type. The experimental trend is reproduced in Fig. 3, which will be discussed later. In addition to population growth, competition was shown to influence the dynamics of the cell cycle. For wild-type cells, competition altered the distribution of cells across different phases of the cell cycle. In the experiments (23), using the FUCCI2 reporter (96), the fraction of wild-type cells in G1 and those entering differentiation (G0 phase) increased in mixed organoids. To quantify the proportion of cells in the S/G2/M phases, EdU and pH3 labeling were also employed, and both methods consistently showed a reduction in the fraction of wild-type cells in these phases in mixed organoids compared with pure organoids. These trends are reproduced in Fig. 4, and will be discussed further in the context of our modeling.

## A Mean-Field Model for the Cell Competition

We focus on identifying the simplest competition scenario that can account for all the above experimental observations while relying on biologically reasonable assumptions. We propose that cells compete for one common environmental resource. However, this resource cannot be nutrients alone. If cancer cells were simply superconsumers of nutrients responsible for depletion in mixed organoids, the depletion would occur in pure cancer population as well, resulting in a detectable fall in the slope of their growth, which is not observed in the experiment (23). Instead, both pure populations exhibit sustained exponential growth throughout the experimental time window. This leads us to conclude that the pure populations grow in a self-sufficient manner. Specifically, if a crucial resource constitutes the competition bottleneck underlying these asymmetric population dynamics, it must be produced by the cells themselves; otherwise, exponential growth would not be observed under pure conditions for either cell type. In addition, the asymmetric variations in populations show a bidirectional interaction that should be due to competition rather than only the presence of some ligands. This points toward competition for autocrine/paracrine signaling ligands. In this mode of signaling, cells secrete molecules such as growth factors, which diffuse through the surrounding environment and act on neighboring cells as well as the secreting cell itself. Secreted growth factors, in turn, promote cell proliferation. Liver progenitor cells (97–102) and colorectal cancer cells (103–105) are known to secrete growth factors as autocrine or paracrine signaling to support their own proliferation.

### Model Formulation.

Our modeling approach is as follows. For each cell, we represent the position in the cell cycle by a quantity called the “cell phase” as a function of time, which we denote by ϕ(t). The value of ϕ ranges from 0 (for a newborn cell) to 2π (for a cell about to divide). Without loss of generality, we may assign the interval [0,π] to the G1 phase and [π,2π] to the remaining phases of the cell cycle, comprised of phases S, G2, and M phases, which we denote by subscript “S” for simplicity. After a cell divides and passes ϕ=2π, its daughter cells restart cycling with ϕ=0. We denote the progression speeds in G1 and in the rest of the cycle by $v_{\text{G1}}$ and $v_{\text{S}}$, respectively.

Instead of tracking single cells in the phase domain, we look at the ϕ-space density of cells $\rho^{x}\left(\phi ,t\right)$, where the symbol x∈{W,C} denotes the cell type, corresponding to wild-type and cancer cells, respectively. We use this notation throughout the text whenever both cell types are represented within the same equation. The total populations of wild-type and cancer cells are indicated using W(t) and C(t), respectively, which are found by $W\left(t\right)=W_{\text{D}}\left(t\right)+\int_{0}^{2\pi }\rho^{W}\left(\phi ,t\right)d\phi$ and $C\left(t\right)=\int_{0}^{2\pi }\rho^{C}\left(\phi ,t\right)d\phi$. We denote the number of differentiated wild-type cells using $W_{\text{D}}\left(t\right)$. The differentiation process for liver progenitor cells in culture takes several days (23). Here, “differentiated” refers to cells that have already entered the G0 phase and have initiated the differentiation process. In the experiments (23), transition into the G0 phase is unidirectional and necessarily leads to differentiation. Therefore, the terms “G0 state” and “differentiated state” are used interchangeably.

It is accepted that cycling cells undergo regulatory commitment to either exit the cell cycle by entering the G0 phase or continue progressing, prior to reaching a specific point in mid-to-late G1 phase (106–112). This point is known as the “restriction point” (R-point). Beyond the R-point, cells are committed to proceed through the subsequent stages of the cell cycle. In this work, we assume the R-point is at the end of the G1 phase. We introduce a transition rate, $r_{D}$, representing the rate at which cells exit G1 and enter the G0 state for differentiation. This rate governs the differentiation of cells prior to reaching the R-point. Naturally, once cells have passed the R-point, no such differentiation occurs.

We make several reducing assumptions to make the model tractable. In summary, these are as follows: i) Each cell secretes a growth factor with a constant rate, S. This can be dependent on the cell type ($S_{W}$ and $S_{C}$), but here we assume them to be equal. ii) Cells consume the growth factor at a rate proportional to its concentration in the environment. However, the proportionality factor for the cancer cells is higher than that for the wild-type cells. This means that, at the same concentration of growth factor, cancer cells consume it at a higher rate than wild-type cells. This assumption is supported by the fact that colorectal cancer cells (113), as other cancer cells of epithelial origin (103) are known to overexpress growth factor receptors on their membranes. Thus, if we denote the growth factor concentration with α, the consumption rates for the wild-type and cancer cells are $\mu_{\text{W}}\alpha \left(t\right)$ and $\mu_{\text{C}}\alpha \left(t\right)$, respectively, where $\mu_{\text{C}}>\mu_{\text{W}}$ are proportionality factors. iii) To avoid geometrical complications, we approximate the concentration of the growth factor by its total amount per capita, α(t)=M(t)/N(t), where M(t) and N(t) are the instantaneous values for the total mass of the growth factor and the total population, respectively.

Growth factors play a significant role in the regulation of the G1 phase (114–117), which includes modification of G1 length (117, 118). The duration of G1 phase has been shown to be shortened at higher concentrations of growth factors (117–119). Therefore, as the next assumption, iv) the speed of cell cycle progression in the G1 phase, $v_{\text{G1}}$, is considered sensitive to the growth factor consumption rate. Specifically, $v_{\text{G1}}^{\text{W}}$ and $v_{\text{G1}}^{\text{C}}$ are assumed to be proportional to the growth factor consumption rates:

[1] $$\begin{array}{c} v_{\text{G1}}^{x}\left(t\right)\;=\;F_{0}\;\mu_{x}\;\alpha \left(t\right). \end{array}$$

The factor $F_{0}$ is a constant that converts the units of consumption rate (kg/h) to those of progression speed ($h^{-1}$). Although more complicated choices like saturation kinetics are possible, we adopt the simplest approach that still captures the growth factor’s promoting effect—namely, a linear dependence. v) Unlike G1, the progression speed, $v_{\text{S}}$, is constant in the phases S/G2/M and independent of growth factor concentration. This is supported by the presence of an R-point in the cell cycle, and by the observation that phases other than G1 show less variability in length (120, 121).

A key assumption vi) underlying the use of a mean-field model, is that the secreted substance rapidly becomes a homogeneously distributed “common good” on time scales much shorter than those of cell division. This assumption was motivated by the geometry of the organoids and the presence of metabolic fluid in their interior, which supports rapid mixing of the secreted substance. A schematic illustration of this is provided in the graphical abstract of the original experimental paper (23). It is easy to justify this assumption by estimating the time scale of diffusion of secreted factors. The diffusion coefficient of growth factors can be estimated using the Stokes–Einstein relation, $D=k_{B}T/\left(6\pi \eta R\right)$. Growth factors are typically proteins with molecular sizes (R) on the order of a few nanometers. Assuming a temperature of T=310K and the viscosity of water (η≈0.001Pa.s), the diffusion coefficient for growth factors falls in the range of approximately $1\times 10^{-11}$ to $1\times 10^{-10}\;{\text{m}}^{2}/\text{s}$. Considering the length scale of organoids to be L≈200μm, this leads to a diffusion time scale $\tau ∼L^{2}/\left(6D\right)$ in the range of 100 to 1,000s. This provides an estimate of the diffusion time, which may be longer in the presence of macromolecules, yet is still expected to remain much shorter than the typical cell-division time (∼20 h).

**Fig. 2.**
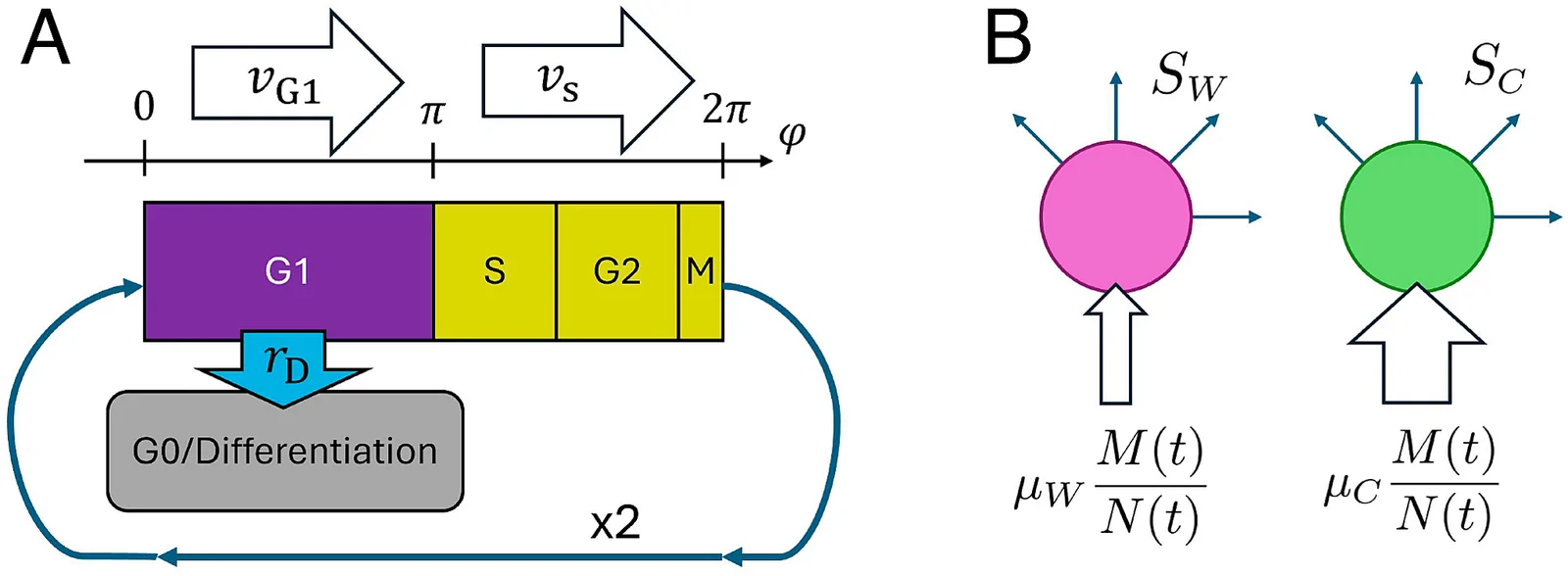
Graphical summary of the modeling approach. (*A*) Schematic representation of the cell cycle used in our mean-field modeling. The G1 phase is shown in purple, and the rest of the cell cycle in yellow. The gray box indicates the wild-type cells that have exited their cycle to start the differentiation process. Differentiation can happen from any point in G1, as indicated using the blue arrow. The axis indicates how the cell phase ϕ changes as a cell progresses through G1 with speed $v_{\text{G1}}$; and through S, G2, and M phases with speed $v_{\text{S}}$. Each time a cell exits the M phase (ϕ=2π), it is doubled and both daughters reenter the G1 phase with ϕ=0. (*B*) Visualization of the assumptions underlying the secretion and the uptake rates in the mean-field model. The wild-type (magenta) and cancer cells (green) are shown as well as their secretion rates indicated by $S_{W}$ and $S_{C}$, respectively. The uptake rates are proportional to the total amount of growth factor per capita with proportionality factors $\mu_{W}$ and $\mu_{C}$ for wild-type and cancer cells where $\mu_{C}$ is assumed to be greater than $\mu_{W}$, as indicated using the width of the arrow. Note that the symbol S in panel (*A*) denotes the “synthesis” phase in the cell cycle and is used only as a label, while the S in panel (*B*) and throughout the equations in the text, represents the source term and is a quantitative variable.

Last, in the experiments, no apoptosis was observed. Therefore, as the assumption vii), we do not include any apoptosis or death rate term in the model. The model assumptions for the cell cycle and the secretion and consumption processes are graphically illustrated in Fig. 2.

Under the above assumptions, the evolution equations for the wild-type and cancer cells read

[2] $$\begin{array}{c} \frac{\partial \rho^{x}}{\partial t}=-\frac{\partial }{\partial \phi }\left(\rho^{x}v^{x}\right)-r^{x}\rho^{x}, \end{array}$$

which represent an advection-reaction system and x∈{W,C}. The differentiation rate r is zero for the cancer cells for all ϕ, while for the wild-type cells it is constant ($r_{\text{D}}$) for ϕ<π and zero otherwise. The progression speed, $v^{x}$, should be replaced by $v_{\text{G1}}^{x}$ for ϕ<π and by $v_{\text{S}}$ for ϕ>π, both being constant with respect to ϕ in the pertaining intervals. However, $v_{\text{G1}}^{x}$ is in general a function of time, while $v_{\text{S}}$ is constant also over time. The boundary conditions for Eq. **2**, are given by flux continuity at ϕ=π, and doubling at ϕ=2π, where the division occurs:

[3] $$\begin{array}{c} v_{G1}^{x}\left(t\right)\;\lim_{\phi \to \pi^{-}}\rho^{x}\left(\phi ,t\right)=v_{S}\;\lim_{\phi \to \pi^{+}}\rho^{x}\left(\phi ,t\right), \end{array}$$

and

[4] $$\begin{array}{c} v_{G1}^{x}\left(t\right)\;\rho^{x}\left(0,t\right)=2\;v_{S}\;\rho^{x}\left(2\pi ,t\right). \end{array}$$

In the following, for brevity, we denote the two limits in Eq. **3** by $\rho^{x}\left(\pi^{-},t\right)$ and $\rho^{x}\left(\pi^{+},t\right)$. For the total amount of the growth factor, M(t), which according to Eq. **1** determines $v_{G1}^{x}$, we obtain:

[5] $$\begin{array}{cc} \frac{\mathrm{dM}}{\mathrm{dt}} & =\;(S-\mu_{\text{W}}\;\frac{M}{N})\;W\left(t\right)\;+\;(S-\mu_{\text{C}}\;\frac{M}{N})\;C\left(t\right), \end{array}$$

where N(t)=W(t)+C(t), is the total population of the organoid. Finally, for the population of wild-type cells going into differentiation, $W_{\text{D}}\left(t\right)$, we have

[6] $$\begin{array}{c} \frac{dW_{\text{D}}}{\mathrm{dt}}=\;r_{\text{D}}\int_{0}^{\pi }\rho^{\text{W}}\left(\phi ,t\right)\;d\phi . \end{array}$$

In Eqs. **1**–**6**, the parameters $r_{D},v_{S},F_{0},\mu_{W},\mu_{C}$, and S appear. To simplify the system and reduce the number of parameters, we nondimensionalized the equations. We used $1/\beta_{W}$ and $1/F_{0}$ as the characteristic time and mass, respectively. Hence, using a tilde to denote dimensionless quantities, we define $\tilde{t}=\beta_{W}t,\;\tilde{M}=F_{0}M,\;{\tilde{r}}_{D}=r_{D}/\beta_{W},\;{\tilde{v}}_{S}=v_{S}/\beta_{W},\;\tilde{\mu }=\mu /\beta_{W},\;\text{and}\;\tilde{S}=SF_{0}/\beta_{W}$, where μ carry a subscript either W or C, indicating wild-type or cancer cells, respectively. Therefore, we can express Eqs. **1**–**6** in dimensionless form, for which we provide solutions.

### Solution for Pure Conditions.

For pure conditions of both types, we assume that the distribution of cells in ϕ is already in quasi-steady state. This means for both types, $\partial \left(\rho \left(\phi ,\tilde{t}\right)/N(\tilde{t})\right)/\partial \tilde{t}=0$, and for wild-type cells, the differentiated fraction, $f_{\text{D}}=W_{\text{D}}/W$ is constant, i.e., $\partial \left(W_{\text{D}}/W\right)/\partial \tilde{t}=0$. The motivation for this choice comes from the exponential growth of both types under pure conditions, which implies a constant proliferation rate. This, in turn, suggests that cells experience similar effective conditions across generations. The solution for the pure wild-type population is

[7] $$\begin{array}{c} \rho^{W}\left(\phi ,\tilde{t}\right)=\left\{\begin{array}{c} \rho^{W}\left(0,0\right)\;\exp\left(-\frac{1+{\tilde{r}}_{D}}{{\tilde{\mu }}_{W}{\tilde{\alpha }}_{W}}\phi \right)\;e^{\tilde{t}},\;\phi \in \left(0,\pi \right),\\ \rho^{W}\left(\pi^{+},0\right)\;\exp\left(-\frac{\phi -\pi }{{\tilde{v}}_{S}}\right)\;e^{\tilde{t}},\;\;\phi \in \left(\pi ,2\pi \right); \end{array}\right. \end{array}$$

[8] $$\begin{array}{c} W_{D}\left(\tilde{t}\right)=f_{D}\;W\left(0\right)\;e^{\tilde{t}}; \end{array}$$

[9] $$\begin{array}{c} \tilde{M}\left(\tilde{t}\right)={\tilde{\alpha }}_{W}\;W\left(0\right)\;e^{\tilde{t}}, \end{array}$$

where ${\tilde{\alpha }}_{W}=\tilde{S}/\left(1+{\tilde{\mu }}_{W}\right)$ is the equilibrium concentration for pure wild-type population. However, the boundary conditions [**3**] and [**4**], together with conditions $\int_{0}^{2\pi }\rho^{W}\left(\phi ,0\right)d\phi =\left(1-f_{D}\right)W\left(0\right)$, $dW_{D}/d\tilde{t}={\tilde{r}}_{D}\int_{0}^{\pi }\rho^{W}\left(\phi ,\tilde{t}\right)d\phi$, and the fact that in experiments $f_{D}\approx 0.2$, reduce the problem to two free parameters, $\tilde{S}$ and ${\tilde{v}}_{S}$. The interdependencies among our equations allow us to obtain the other quantities, see *SI Appendix*.

[10] $$\begin{array}{c} {\tilde{r}}_{D}=f_{D}/\left(2-f_{D}-e^{\pi /{\tilde{v}}_{S}}\right); \end{array}$$

[11] $$\begin{array}{c} {\tilde{\mu }}_{W}=1/\left(\tilde{S}m/\left(1+{\tilde{r}}_{D}\right)-1\right); \end{array}$$

[12] $$\begin{array}{c} \rho^{W}\left(0,0\right)=2m\left(1-f_{D}\right)W\left(0\right)/\left(1+{\tilde{r}}_{D}\left(e^{\pi /{\tilde{v}}_{S}}-1\right)\right); \end{array}$$

and

[13] $$\begin{array}{c} \rho^{W}\left(\pi^{+},0\right)=\rho^{W}\left(0,0\right)\left({\tilde{\mu }}_{W}{\tilde{\alpha }}_{W}/{\tilde{v}}_{S}\right)\;e^{-m\pi }, \end{array}$$

where for simplicity, we introduce $m=\left(1+{\tilde{r}}_{D}\right)/\left({\tilde{\mu }}_{W}{\tilde{\alpha }}_{W}\right)=\;\ln2/\pi -1/{\tilde{v}}_{S}$. Thus, the solution for pure wild-type population is complete once $\tilde{S}$ and ${\tilde{v}}_{S}$ are given.

For pure cancer cells, where differentiation is assumed absent, we have

[14] $$\begin{array}{c} \rho^{C}\left(\phi ,\tilde{t}\right)=\left\{\begin{array}{cc} \rho^{C}\left(0,0\right)\;\exp\left(-\frac{\gamma }{{\tilde{\mu }}_{C}{\tilde{\alpha }}_{C}}\;\phi \right)\;e^{\gamma \tilde{t}} & ,\phi \in (0,\pi );\\ \rho^{C}\left(\pi^{+},0\right)\;\exp\left(-\frac{\gamma (\phi -\pi )}{{\tilde{v}}_{S}}\right)\;e^{\gamma \tilde{t}} & ,\phi \in (\pi ,2\pi ); \end{array}\right. \end{array}$$

[15] $$\begin{array}{c} \tilde{M}\left(\tilde{t}\right)={\tilde{\alpha }}_{C}\;C\left(0\right)\;e^{\gamma \tilde{t}}, \end{array}$$

where $\gamma =\beta_{C}/\beta_{W}$, and ${\tilde{\alpha }}_{C}=\tilde{S}/\left(\gamma +{\tilde{\mu }}_{C}\right)$ is the equilibrium concentration for pure cancer cells. Again, assuming $\tilde{S}$ and ${\tilde{v}}_{S}$ are given, we use boundary conditions [**3**] and [**4**] as well as $\int_{0}^{2\pi }\rho^{C}\left(\phi ,0\right)d\phi =C\left(0\right)$, to obtain ${\tilde{\mu }}_{C}$, $\rho^{C}\left(0,0\right)$ and $\rho^{C}\left(\pi^{+},0\right)$:

[16] $$\begin{array}{c} {\tilde{\mu }}_{C}=\gamma^{2}/\left(\tilde{S}q-\gamma \right); \end{array}$$

[17] $$\begin{array}{c} \rho^{C}\left(0,0\right)=2qC\left(0\right); \end{array}$$

and

[18] $$\begin{array}{c} \rho^{C}\left(\pi^{+},0\right)=C\left(0\right)\;\left(\gamma /{\tilde{v}}_{S}\right)\;e^{\gamma \pi /{\tilde{v}}_{S}}, \end{array}$$

where, for brevity, we define $q=\gamma /\left({\tilde{\mu }}_{C}{\tilde{\alpha }}_{C}\right)=\;\ln2/\pi -\gamma /{\tilde{v}}_{S}$. Since we are interested in the normalized populations, we set W(0)=1 and C(0)=1 for pure conditions. Populations calculated from densities above, are plotted in Fig. 1*A* as well as the experimental data. As is obvious from the equations, in the quasi-steady state, cells are exponentially distributed in ϕ, and the densities at any ϕ increase exponentially with time, reproducing the same growth behavior as we see in the experiment. Constant values of growth factors per capita (${\tilde{\alpha }}_{W}$ and ${\tilde{\alpha }}_{C}$) here, arise from the quasi–steady-state assumption. Full derivations are presented in *SI Appendix*.

### Solution for Mixed Conditions.

The solutions we previously presented for pure organoids guarantee the exponential growth shown in Fig. 1*A* and ensure that the fraction of differentiating cells remains equal to $f_{D}$ in pure conditions, regardless of the values of $\tilde{S}$ and ${\tilde{v}}_{S}$. For the mixed organoids, we numerically solve Eq. **2** for both types, as well as Eqs. **5** and **6** using the boundary conditions provided in Eqs. **3** and **4**. The initial cell counts for mixed organoids are determined by drawing random samples from synthetic distributions fitted to experimental data. Specifically, we fitted exponential distributions in the form $f\left(n\right)=\left(1/\theta \right)\;e^{-(n-n_{0})/\theta }$ to the initial cell numbers in the experiments, and sampled randomized values to serve as initial conditions for the model. For wild-type and cancer cells, the distribution parameters turned out to be $\theta_{\text{W}}=53.0,\;n_{0}^{\text{W}}=14.0,\;,\theta_{\text{C}}=38.2\;\text{and}\;n_{0}^{\text{C}}=2.0$. Full details of the distribution fitting process are provided in *SI Appendix*. From these distributions, 500 pairs of random samples were drawn to serve as the initial counts of each cell type in mixed organoids. Once the initial counts of wild-type and cancer cells are established, the initial concentration of growth factors is computed as a weighted average of the equilibrium concentrations, which is

[19] $$\begin{array}{c} \tilde{M}\left(0\right)=N\left(0\right)\;{\tilde{\alpha }}_{\text{avg}}=W\left(0\right)\;{\tilde{\alpha }}_{W}+C\left(0\right)\;{\tilde{\alpha }}_{C}. \end{array}$$

Thus far, $\tilde{S}$ and ${\tilde{v}}_{S}$ are assumed given. To fully solve the problem, these parameters must therefore be identified. Their values are obtained by minimizing a cost function that penalizes the discrepancy between the model predictions and the experimental data. The cost function comprises three terms which account for the populations in mixed organoids, the statistics of wild-type cells, and the effects of initial percentage of cells. It is expressed as

[20] $$\begin{array}{c} L\left(\tilde{S},{\tilde{v}}_{S}\right)=\omega_{\text{p}}L_{\text{pop}}+\omega_{\text{s}}L_{\text{stat}}+\omega_{\text{i}}L_{\text{init}}. \end{array}$$

The terms $L_{\text{pop}}$, $L_{\text{stat}}$, and $L_{\text{init}}$ represent the errors for population dynamics, cell statistics in different phases, and the effect of initial composition, respectively. They are the weighted squared errors between the experimental data and the predictions of the model. The factors $\omega_{\text{p}}$, $\omega_{\text{s}}$, and $\omega_{\text{i}}$ are used to weight different terms and, for simplicity, are all considered 1/3. Full details on calculation of each term are presented in *SI Appendix*.

## Results

In this section, we first present the model predictions obtained with the best-fit parameter set and compare them with the experimental observations. The independent parameters were found to be $\tilde{S}=19.6\;\pm \;0.2$ and ${\tilde{v}}_{S}=10.8\;\pm \;0.1$. The biophysical meanings of these numerical values follow from their definitions in *Model Formulation*: $\tilde{S}=S/S_{0}$ represents the ratio of the secretion rate to a reference mass rate, $S_{0}=\beta_{W}/F_{0}$. Here, $\beta_{W}$ denotes a rate ($\beta_{W}=\ln2/\tau_{d}^{W}$), while $1/F_{0}$ defines growth factor consumption per unit phase progression, as given by Eq. **1**. If we denote the total growth factor consumption during the G1 phase by $\Delta M_{G1}$, we obtain $S_{0}=\left(\ln2/\pi \right)\Delta M_{G1}/\tau_{d}^{W}$. In addition, ${\tilde{v}}_{S}$ directly yields $v_{S}=\beta_{W}{\tilde{v}}_{S}$, which in turn gives the duration of the S/G2/M phases as $T_{S}=\pi /v_{S}=10.1\;h$. Further interpretation will be provided in *Discussion*. From these, the remaining parameters followed ${\tilde{\mu }}_{W}=1.34\pm 0.05$, ${\tilde{\mu }}_{C}=5.24\pm 0.4$, ${\tilde{\alpha }}_{W}=8.37\pm 0.3$, ${\tilde{\alpha }}_{C}=2.94\pm 0.2$, and ${\tilde{r}}_{D}=0.43\pm 0.005$. A comparison between ${\tilde{\mu }}_{W}$ and ${\tilde{\mu }}_{C}$ indicates that the growth factor consumption capacity of cancer cells is approximately four times greater than that of wild-type cells. Using the obtained numerical values, the densities $\rho^{x}\left(\phi ,t\right)$ and the total populations W(t) and C(t) were found. The resulting populations overlay the experimental data from ref. 23 for both pure and mixed organoids, as shown in Fig. 1*A*. In pure conditions, the growth curves are exponential, as expected from the analytical solutions. In the mixed conditions, the growth rate of wild-type cells decreases, whereas that of cancer cells increases.

The variations in the populations shown in Fig. 1*A*, can be explained by Fig. 1*B* of the same figure. In this panel, we have plotted the dimensionless G1-progression speed, ${\tilde{v}}_{G1}$, as a function of dimensionless growth factor per capita, $\tilde{\alpha }$, for wild-type and cancer cells, as described in the original form, by Eq. **1**. The curve for cancer cells has a higher slope due to their higher uptake capacity. This allows cancer cells to have a higher G1 progression speed than wild-type cells, not only at any equal concentration of growth factor α, but also at lower values (down to $(\mu_{\text{W}}/\mu_{\text{C}})\alpha$). It can be understood by comparing the equilibrium concentrations of pure organoids, shown by solid circular markers in Fig. 1*B*. In mixed organoids, the growth factor concentration lies between the two extremes observed in pure wild-type and pure cancer populations. An example value is shown by open square markers in Fig. 1*B*. Notably, the different slopes for wild-type and cancer cells in Fig. 1*B* lead to an asymmetry in how ${\tilde{v}}_{G1}$ varies for the two types. That is, mixing exposes wild-type cells to a lower concentration than in their pure conditions, resulting in a reduced ${\tilde{v}}_{G1}$. In contrast, cancer cells are exposed to a higher concentration than in pure conditions, leading to an increase in ${\tilde{v}}_{G1}$. This characteristic consequently leads to the asymmetric variations in populations, which is shown in Fig. 1*A*. There is, however, a slight mismatch between the model and the experiment for cancer cells in mixed organoids in the interval 10∼30h. This is because, for a number of the mixed organoids, the population of cancer cells was observed to decrease in this interval.

**Fig. 3.**
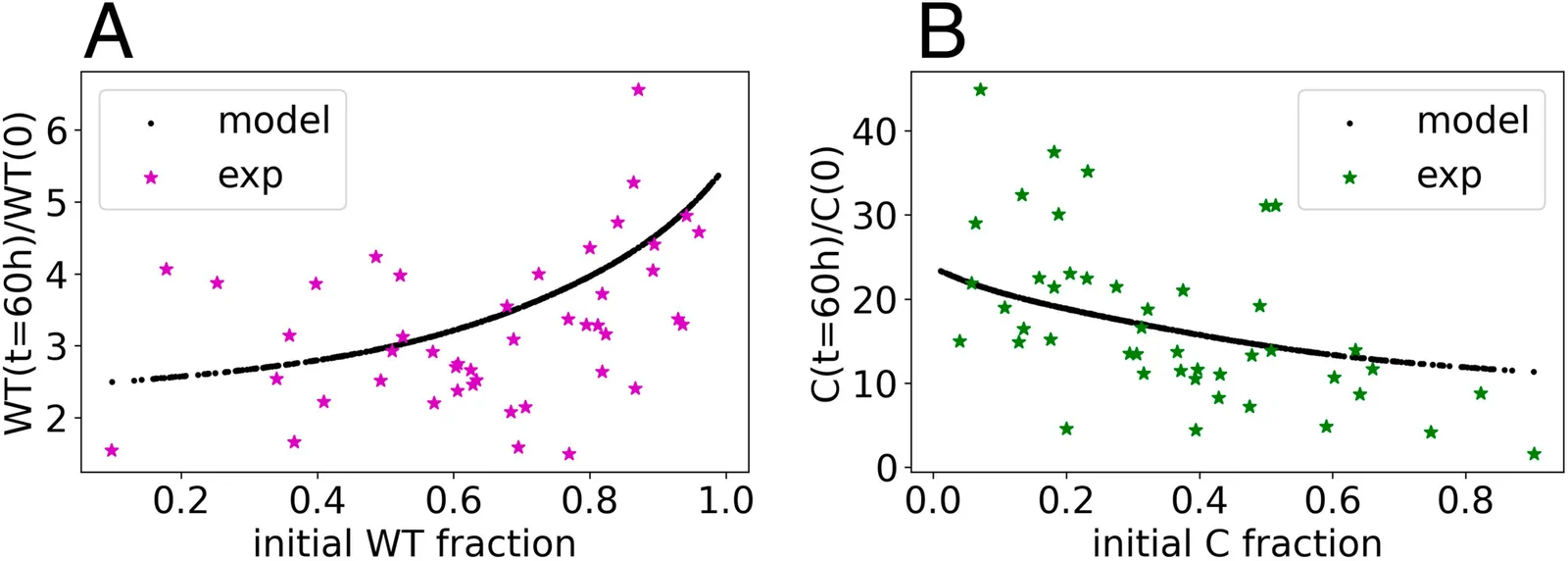
Normalized population growth of each cell type at t=60 h vs. their initial fraction in the mixture. The data are shown for (*A*) wild-type cells and (*B*) cancer cells. The stars show the data from the experiment, while the dots from the model. The number of organoids for the experimental data is n=45 and for the model, n=500.

Next, we considered the impact of the initial composition of the organoid. Following the experimental workflow, we plotted the normalized population size of each cell type at t=60h vs. its initial fraction in the mixed organoids. The resulting data of the model, together with the experimental data, are shown in Fig. 3. We capture the same trend of the experiments: The growth of wild-type cells increases with their starting proportion, whereas the growth of cancer cells decreases as their own starting proportion rises. However, the match between the trend in model and experiment is better for wild-type cells than for cancer cells.

**Fig. 4.**
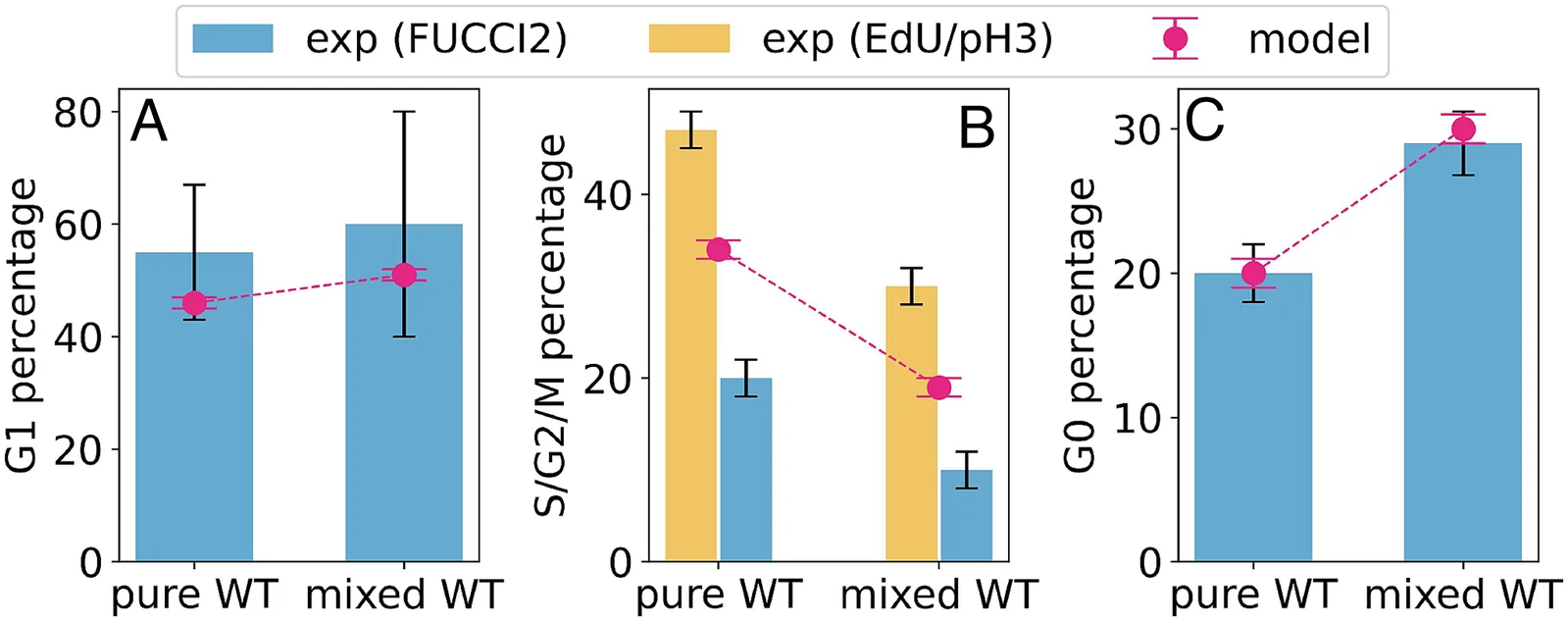
Comparison of experimental data and model predictions for the proportions of wild-type cells in (*A*) G1 phase, (*B*) S/G2/M phases, and (*C*) G0 phase of the cell cycle. The data are provided for pure and mixed organoids at t=60h. The experimental data are taken from ref. 23. For the cells in S/G2/M phases, the two different experimental measurement methods are shown. Uncertainties for experimental data indicate SEM. For the model, a basic 1% uncertainty due to rounding was considered. The statistical SEM was smaller. The dashed lines connect the data of the model to guide the eye.

Competition alters the distribution of wild-type cells across cell cycle phases, as observed experimentally. We have made a comparison of the percentages of cells in different phases between model and experiment. Fig. 4 presents the percentages of wild-type cells in each phase of the cell cycle obtained from the experiment and the model at t=60h, the only time point for which experimental data are available. The model reproduces the proportions G0 and G1 within the corresponding experimental uncertainties. For the S/G2/M phases, two complementary assays were employed, as described in the section on experimental observations: FUCCI2 reporter and combined EdU + pH3 protocol. Averaging these two measurements yields a balanced reference value which was used as the target value in the cost minimization. This is what the model reproduces, as shown in the *Middle* panel of Fig. 4. Most importantly, the mean-field formulation captures every experimentally observed shift in phase distribution that occurs when wild-type cells reside in mixed organoids compared to the pure conditions. As a complementary analysis, we studied the model’s predictions for the temporal evolution of phase-specific cell fractions, which is discussed in *SI Appendix*.

To evaluate the robustness of the model, we studied the sensitivity of its predictions to the parameters $\tilde{S}$ and ${\tilde{v}}_{S}$. This was done by solving the problem for a range of values of the two parameters, which covered almost ±5% of the fitted values. We first ensured that the captured critical values for $\tilde{S}$ and ${\tilde{v}}_{S}$ define a global minimum in the landscape of cost function, by analyzing the Hessian matrix. Next, we found the directions defining the highest and the lowest curvature of the cost function using eigenvectors of the Hessian matrix, which were $\tilde{S}-{\tilde{S}}^{\text{fit}}=0.32\left({\tilde{v}}_{\text{S}}-{\tilde{v}}_{\text{S}}^{\text{fit}}\right)$ and $\tilde{S}-{\tilde{S}}^{\text{fit}}=-3.1\left({\tilde{v}}_{\text{S}}-{\tilde{v}}_{\text{S}}^{\text{fit}}\right)$, respectively. This shows that the direction for the least sensitivity aligns closer to the direction of $\tilde{S}$, while sensitivity is higher in the direction of ${\tilde{v}}_{S}$. The reason for this anisotropy is that the variation in ${\tilde{v}}_{S}$ controls the fitting process in two ways. First, it determines the fractions of cells in S/G2/M, which in turn contributes to $L_{\text{stat}}$ in the cost function. Furthermore, the fractions of division time between the S/G2/M phases and the G1 phase are controlled by ${\tilde{v}}_{S}$. Since the G1 phase is the window for competition, ${\tilde{v}}_{S}$ also indirectly controls population growth, contributing to $L_{\text{pop}}$. In contrast, the model shows less dependence on the secretion term, $\tilde{S}$. This means that the model predictions would remain qualitatively unchanged with slightly different secretion rates and even with unequal secretion rates for wild-type and cancer cells. See *SI Appendix*, *Sensitivity Analysis* for a detailed discussion.

**Fig. 5.**
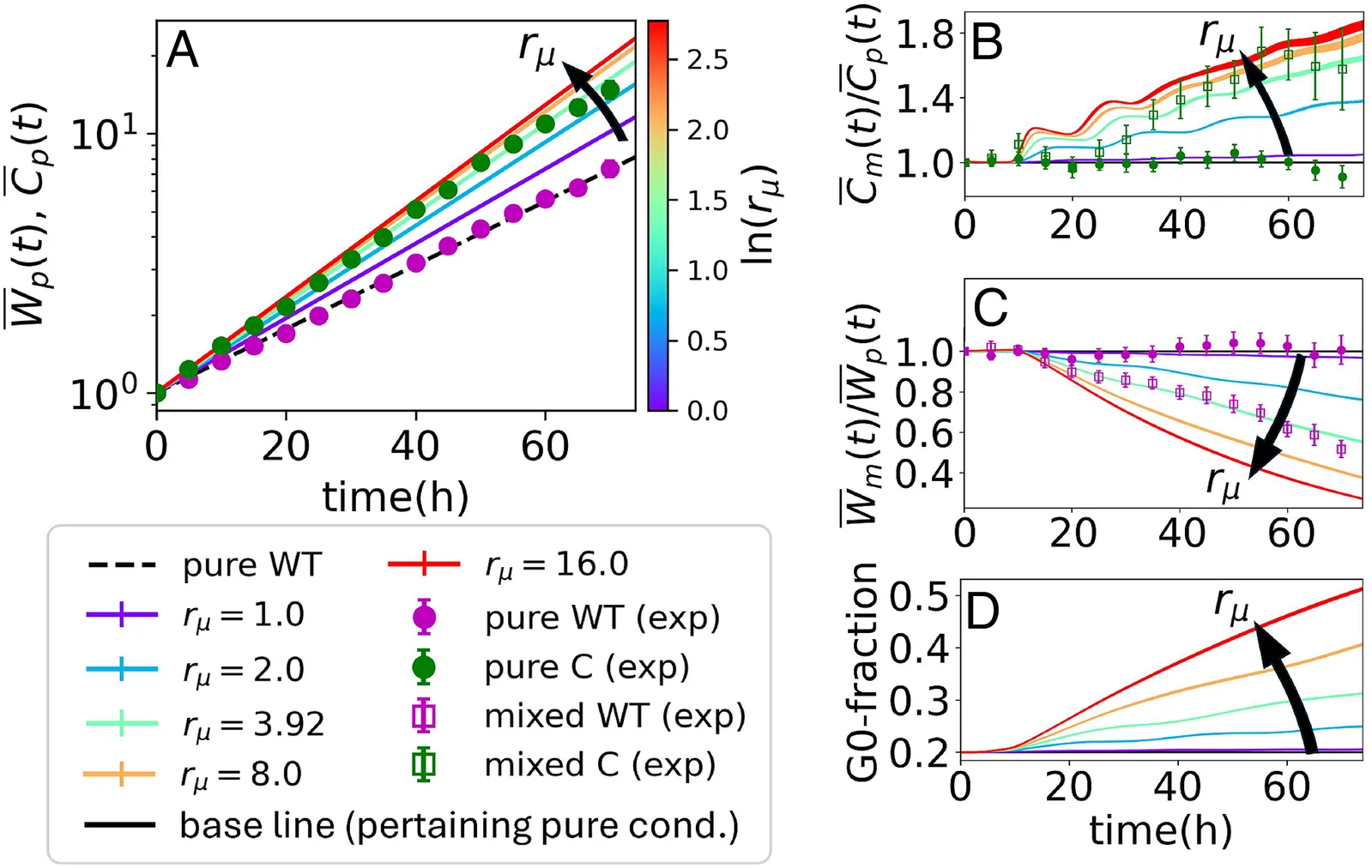
Influence of uptake coefficient ratio on competition. (*A*) Normalized populations under pure conditions for both cell types at different values of $r_{\mu }={\tilde{\mu }}_{C}/{\tilde{\mu }}_{W}$. (*B* and *C*) Ratios of normalized populations in mixed and pure conditions for (*B*) cancer and (*C*) wild-type cells as a function of time. (*D*) Fraction of differentiated wild-type cells (G0 phase) as a function of time for different values of $r_{\mu }$. Colors in all panels correspond to the color bar in panel (*A*). The arrows show the direction along which $r_{\mu }$ increases. The legend applies to all panels. Uncertainties in all panels indicate SEM.

The difference between the uptake coefficient factors ${\tilde{\mu }}_{W}$ and ${\tilde{\mu }}_{C}$ is a key factor resulting in the asymmetric variation of growth curves shown in Fig. 1*A*. The parameter fitting yields a ratio $r_{\mu }={\tilde{\mu }}_{C}/{\tilde{\mu }}_{W}=3.92$ for the coefficients. To investigate how this ratio influences the competition, we varied $r_{\mu }$ by adjusting ${\tilde{\mu }}_{C}$ while keeping all other parameters, including ${\tilde{\mu }}_{W}$, fixed. The ratio $r_{\mu }$ is swept from 1 to 16, which encompasses the fitted value of 3.92, and the resulting model behavior is shown in Fig. 5. First, the normalized populations of wild-type and cancer cells under pure conditions are shown in Fig. 5*A*. Since ${\tilde{\mu }}_{W}$ is kept constant, the pure wild-type population remains unchanged, whereas the pure cancer population increases with $r_{\mu }$. Even at $r_{\mu }=1$, a difference exists between cancer and wild-type cells: A fraction of wild-type cells continually initiate differentiation and cease proliferating, while cancer cells are assumed not to differentiate at all. In the next step, we examined the level of competition by considering two quantities. The first is the ratio of the normalized populations in mixed and pure conditions for each type, which indicates how strongly population growth deviates from the pure conditions as a result of competitive interactions. The second is the differentiated fraction of wild-type cells, representing the proportion of wild-type cells that have exited the cell cycle. In panels (*B* and *C*) of Fig. 5, we plot the ratios ${\bar{W}}_{m}\left(t\right)/{\bar{W}}_{p}\left(t\right)$ and ${\bar{C}}_{m}\left(t\right)/{\bar{C}}_{p}\left(t\right)$, where $\bar{W}$ and $\bar{C}$ denote the normalized populations of wild-type and cancer cells, and the subscripts *m* and *p* refer to mixed and pure conditions, respectively. As shown in Fig. 5*B* and *C*, this ratio increases for cancer cells and decreases for wild-type cells as $r_{\mu }$ increases. We further investigated the interplay of $r_{\mu }$ and ${\tilde{r}}_{\text{D}}$ in *SI Appendix*. Our analysis showed that the ratio $r_{\mu }$ plays a more important role than ${\tilde{r}}_{\text{D}}$ in regulating the quantities ${\bar{W}}_{m}\left(t\right)/{\bar{W}}_{p}\left(t\right)$ and ${\bar{C}}_{m}\left(t\right)/{\bar{C}}_{p}\left(t\right)$ as the main indicators of competition. Finally, Fig. 5*D* shows the fraction of differentiated wild-type cells in mixed conditions over time for different values of $r_{\mu }$. The results indicate that higher ratios of uptake coefficients accelerate cell cycle exit in wild-type cells. Clearly, competitive interactions intensify as the ratio of uptake coefficients between the two cell types increases.

## Discussion

We derived analytical solutions for pure populations and numerical solutions for mixed organoids using the dimensionless forms of Eqs. **2**, **5**, and **6**. These equations contain parameters that must be determined from experimental data. Assessing whether these parameters can be uniquely and reliably estimated—and with what confidence—is the subject of identifiability analysis (122–126). To establish structural identifiability, we used the analytical expressions in Eqs. **10**, **11**, and **16**, together with numerical sweeps over the parameter space of $\tilde{S}$ and ${\tilde{v}}_{\text{S}}$. We then performed sensitivity and confidence analyses in the same two-dimensional parameter space to determine the 95% confidence region for $\tilde{S}$ and ${\tilde{v}}_{\text{S}}$. A detailed discussion of both analyses and a hierarchical diagram showing the parameter determination process is provided in *SI Appendix*.

The physical meaning of ${\tilde{v}}_{\text{S}}$ is directly related to cell division time which can be determined under pure conditions:

[21] $$\begin{array}{c} T_{\text{d}}^{x}=\pi {\beta_{\text{W}}}^{-1}\left(1/{\tilde{v}}_{\text{G1}}^{x}+1/{\tilde{v}}_{\text{S}}\right), \end{array}$$

where ${\tilde{v}}_{\text{G1}}^{x}$ is evaluated at the quasi–steady-state concentrations for each type, that is, ${\tilde{\mu }}_{x}{\tilde{\alpha }}_{x}$. We find $T_{\text{d,pure}}$ to be 20.1h for wild-type cells and 17.4h for cancer cells. In mixed conditions, as is shown in Fig. 1*B*, we always have ${\tilde{\alpha }}_{C}<\tilde{\alpha }<{\tilde{\alpha }}_{W}$. This consequently defines an upper limit for $T_{\text{d}}^{\text{W}}$, and a lower limit for $T_{\text{d}}^{\text{C}}$, which are

[22] $$\begin{array}{c} T_{\text{d,max}}^{\text{W}}=\pi {\beta_{\text{W}}}^{-1}\left({\left({\tilde{\mu }}_{\text{W}}{\tilde{\alpha }}_{\text{C}}\right)}^{-1}+{\tilde{v}}_{\text{S}}^{-1}\right)=38.3\;\text{h}; \end{array}$$

and

[23] $$\begin{array}{c} T_{\text{d,min}}^{\text{C}}=\pi \beta_{\text{W}}^{-1}\left({\left({\tilde{\mu }}_{\text{C}}{\tilde{\alpha }}_{\text{W}}\right)}^{-1}+{\tilde{v}}_{\text{S}}^{-1}\right)=12.8\;\text{h}. \end{array}$$

These two, respectively, describe situations where a wild-type cell or a cancer cell resides in an infinite background of the other type. The calculated extreme values of the division time, together with those obtained under pure conditions, define the intervals within which the division times under mixed conditions lie. For wild-type cells, the division time in mixed conditions is always longer than that in pure conditions, whereas for cancer cells it is always shorter. In other words, we always have $T_{\text{d,pure}}^{\text{W}}<T_{\text{d,mixed}}^{\text{W}}<T_{\text{d,max}}^{\text{W}}$ and $T_{\text{d,min}}^{\text{C}}<T_{\text{d,mixed}}^{\text{C}}<T_{\text{d,pure}}^{\text{C}}$.

The duration of G1 phase is shown to be a determinant in cell cycle timing (121) and correlates with cell fate (117). Hence, it is informative to determine the fraction of cell division time dedicated to the G1 phase, which in general is

[24] $$\begin{array}{c} g=T_{\text{G1}}/T_{\text{d}}=\left(\pi /{\tilde{v}}_{\text{G1}}\right)/\left(\pi /{\tilde{v}}_{\text{G1}}+\pi /{\tilde{v}}_{\text{S}}\right), \end{array}$$

where ${\tilde{v}}_{\text{G1}}$ for pure wild-type and cancer cells is ${\tilde{\mu }}_{\text{W}}{\tilde{\alpha }}_{\text{W}}$ and ${\tilde{\mu }}_{\text{C}}{\tilde{\alpha }}_{\text{C}}$, respectively. We obtain $g_{\text{W,pure}}=0.49$ and $g_{\text{C,pure}}=0.41$ for wild-type and cancer cells, respectively. Given that G1 phase duration ratio is highly variable, spanning from 0.1∼0.15 in pluripotent stem cells (127) to more than 0.6 in different human cells (128), the values we obtain here appear to be reasonable. Similarly to the division time, we can establish an upper bound for g in wild-type cells, and a lower bound in cancer cells. These correspond to $T_{\text{d,max}}^{\text{W}}$ and $T_{\text{d,min}}^{\text{C}}$, yielding $g_{\text{max}}^{W}=0.73$ and $g_{\text{min}}^{C}=0.20$, respectively. In the limiting case of long-term exposure, the average division time of wild-type cells approaches $T_{\text{d,max}}^{\text{W}}$, while that of cancer cells converges to their intrinsic value, $T_{\text{d,pure}}^{\text{C}}$. This occurs because, under these conditions, the cancer population dominates and α tends toward $\alpha_{C}$. In addition, the elongation of G1 phase in wild-type cells leads to an increase in differentiation rate.

Autocrine secretion rate of growth factors in epithelial cells, can be variable depending on the type of growth factors. For example, it can range from the order ∼10^3^ molecules/h for TGF-α to almost $10^{4}∼10^{5}$ molecules/h for EGF, the uptake rate being of the same order of magnitude (129, 130). In our model, the uptake-to-secretion ratios for wild-type and cancer cells in their pure conditions are ${\tilde{\mu }}_{\text{W}}{\tilde{\alpha }}_{\text{W}}/\tilde{S}\approx 0.57$ and ${\tilde{\mu }}_{\text{C}}{\tilde{\alpha }}_{\text{C}}/\tilde{S}\approx 0.78$, respectively, with the higher value in cancer cells reflecting their enhanced proliferative capacity. In addition, our sensitivity analysis showed that the model’s behavior is robust to variations in the secretion rate $\tilde{S}$.

Our model does not require the growth factors produced by the two cell types to be identical. In practice, multiple growth factors ($S_{1},S_{2},\ldots$) may be present, which we represent by a single effective factor (S). The model only assumes that both cell types secrete combinations of such factors, that these combined signals are beneficial to both populations, that cancer cells effectively uptake the ligands more efficiently, and that for both cell types, G1 progression increases with the overall concentration of these combined factors.

The asymmetric variations in growth curves of the two types are the most insightful feature of the observed competition, which emerged from biased uptake capacities in the model. Our modeling choice of this is based on traits previously reported in the literature, including autocrine/paracrine signaling in liver cells (97–102) and cancer cells (103–105), overexpression of growth factor receptors in cancer cells (103, 113), and the regulatory role of growth factors in the G1 phase of the cell cycle (114–117, 118). The unperturbed exponential growth of pure organoids, as well as the initial composition dependence that is reproduced as natural outcomes of our choices, suggests that our model offers a plausible explanation of the experimentally observed competition.

The G1 phase in cell cycle is the stage where cells integrate division-commitment signals, and its duration is regulated by growth factor concentration (114, 117, 118, 131). Motivated by these findings, we assumed that the progression speed of cells in G1, $v_{G1}$, is—in the simplest case—linearly dependent on their growth factor uptake rates, which in turn implies proportionality to the growth factor concentration. Using this assumption, we reproduced the variations in cell phase distributions observed in wild-type cells. To keep the model minimal, we assumed the simplest dependence of progression speed on cell-cycle phase: a linear dependence on growth factor uptake in G1 (Eq. **1**), and a constant speed in S/G2/M phases ($v_{S}$). Yet, this was sufficient to capture variations in both growth curves and cell-phase distributions. We also considered a step function-like averaging for the initial condition of $\tilde{M}$, based on Eq. **19**. This leads to transient oscillations in the phase fractions in time, which are discussed in *SI Appendix*. Using the methods employed by Krotenberg Garcia and colleagues (23), it was not possible to track these phase fractions in real time, as the measurements were terminal and thus precluded further observation of the same samples. It would be valuable to apply alternative, nonterminal approaches to evaluate these fractions at multiple time points, enabling a more robust assessment of the model’s predictions.

The model presented here describes a biochemical competition scenario and relies on a mean-field approximation. As such, it does not account for spatial effects such as geometry, heterogeneity, microenvironmental influences, or mechanical interactions. These limitations naturally restrict the model’s ability to capture the full extent of the experimental variability—for example, the scatter observed in Fig. 3. Nevertheless, the model succeeds in achieving its goal of identifying a minimal competition framework that robustly reproduces the average trends and behaviors, including the emergence of asymmetric population dynamics and cell-cycle modifications. It should also be noted that the validity of the model in describing cancer invasion in real liver tissue is limited in some aspects by the organoid experiments on which it is based. For instance, immune responses are absent in organoids and are therefore not represented in the model, although they may play an important role in competition in vivo. Despite these limitations, our modeling allowed us to extract the functionalities and perform sensitivity analysis on a broad range of parameters; something that was more limited in the experiments.

Our model incorporates two key features: i) biased competition for autocrine/paracrine signaling ligands and ii) G1-phase sensitivity to those signals. In the broader consumer–resource framework, these translate to i) competition on consumer-produced resources and ii) growth-phase-dependent competitive behavior. Each of these features have been examined separately before. Cell-phase-specific competition has been approached in modeling frameworks (48, 49, 85, 90). In addition, consumer-produced resources have also been studied as a model for agriculture (132) and in the context of metabolic byproducts (45, 133). The model presented here, integrates both of these features in a single framework. Our approach is readily applicable to any competitive community where consumers produce and consume resources, and where their growth response depends on their age or proliferative status. Traditionally, in modeling competition using resource-consumer models, the process of reproduction is considered as a black box which appears as a time-derivative of population in the equations, while the dynamics of growth and reproduction phases can be unequally dependent on competition, as suggested here. Competition in growing agricultural human communities is another example of such a situation. However, modification of the model is needed, if features of sexual reproduction—as opposed to asexual reproduction of cells—are critically important.

Following this work, suggestions for future research can be made. On the experimental side, studies can be continued by making comparative measurements in concentrations of candidate growth factors, or by exposing each cell type to the fluid collected from the other type to measure growth variations. Additionally, monitoring temporal changes in cell-cycle phase distributions would also provide valuable data for testing the model’s predictions. On the modeling side, a natural complementary step is simulating the organoids using cell-based models, which can take spatial dependencies or possible roles of mechanical forces into account. Furthermore, alternative formulations of ligand uptake—such as receptor occupancy determining the G1-to-S transition (134)—could be explored to assess whether the observed dynamics is reproduced under different uptake assumptions.

## Concluding Remarks

In this study, we introduced a minimal mean-field model that proposes a possible mechanism for cell competition. Our model incorporates cell cycle dynamics and unequal uptake capacities for secreted signaling ligands in cell cultures. This is inspired by biased competition observed between liver progenitor cells and colorectal cancer cells (23). In this specific case, the growth stage corresponds to the cell cycle, and the shared, consumer-produced resource is the signaling ligands. To maintain simplicity, we considered a minimal set of elements required to reproduce the experimental data. These include that competition takes place through a single biochemical factor, which is common to all cells due to rapid diffusion. Our analysis revealed that if it is the case, three key elements are necessary to reproduce the experimentally observed outcome: 1) the secretion of the factor by cells in an autocrine/paracrine manner; 2) differences in the ability of cell types to respond to the factor; and 3) cell-cycle-dependent sensitivity to the factor. All three elements have been previously observed in the cell types studied here. We also showed that the strength of competitive interactions is governed by the disparity in the response capacities of the cell types.

The model presented here provides solid foundation for future research. On the modeling side, growth-stage-dependent competition can be further explored not only in the context of cell cultures, but also in sociophysical settings, such as ecological competition in expanding agricultural communities.

## Materials and Methods

The experimental dataset used in this study is identical to that reported in the experimental work upon which the present study is based (23), for details on acquisition we refer to this publication. The mathematical framework and governing equations are presented in *Model Formulation*. Analytical derivations underlying the solutions presented in the manuscript are provided in *SI Appendix*. Numerical solutions were obtained in Python 3 using the NumPy and SciPy libraries. Spatial derivatives were discretized using a finite-difference scheme, and temporal integration was performed using the Euler forward method. The maximum Courant number was maintained below 0.5 to ensure numerical stability. Figures were generated using Matplotlib. Parameter values and initial conditions are provided in the main text and *SI Appendix* where applicable.

## Supplementary Material

Appendix 01 (PDF)

## Data Availability

Codes, numerical data, and parameter files data have been deposited in Yoda (https://doi.org/10.24416/UU01-DA6TUG) (135).

## References

[1] S. M. van Neerven, L. Vermeulen, Cell competition in development, homeostasis and cancer. Nat. Rev. Mol. Cell Biol. **24**, 221–236 (2023). 36175766 10.1038/s41580-022-00538-y

[2] M. E. van Luyk, A Krotenberg Garcia, M Lamprou, SJE Suijkerbuijk, Cell competition in primary and metastatic colorectal cancer. Oncogenesis **13**, 1–18 (2024). 39060237 10.1038/s41389-024-00530-5 PMC11282291

[3] N. E. Baker, Emerging mechanisms of cell competition. Nat. Rev. Genet. **21**, 683–697 (2020). 32778819 10.1038/s41576-020-0262-8 PMC8205513

[4] S. Bowling, K. Lawlor, T. A. Rodríguez, Cell competition: the winners and losers of fitness selection. Development **146**, dev167486 (2019). 31278123 10.1242/dev.167486

[5] T. M. Parker , Cell competition in intratumoral and tumor microenvironment interactions. EMBO J. **40**, e107271 (2021). 34368984 10.15252/embj.2020107271 PMC8408594

[6] A. Krotenberg Garcia , Active elimination of intestinal cells drives oncogenic growth in organoids. Cell Rep. **36**, 109307 (2021). 34233177 10.1016/j.celrep.2021.109307 PMC8278394

[7] S. M. van Neerven , Apc-mutant cells act as supercompetitors in intestinal tumour initiation. Nature **594**, 436–441 (2021). 34079128 10.1038/s41586-021-03558-4

[8] M. K. Yum , Tracing oncogene-driven remodelling of the intestinal stem cell niche. Nature **594**, 442–447 (2021). 34079126 10.1038/s41586-021-03605-0 PMC7614896

[9] D. J. Flanagan , NOTUM from Apc-mutant cells biases clonal competition to initiate cancer. Nature **594**, 430–435 (2021). 34079124 10.1038/s41586-021-03525-z PMC7615049

[10] N. Kedia-Mehta, D. K. Finlay, Competition for nutrients and its role in controlling immune responses. Nat. Commun. **10**, 2123 (2019). 31073180 10.1038/s41467-019-10015-4 PMC6509329

[11] A. R. Freischel , Frequency-dependent interactions determine outcome of competition between two breast cancer cell lines. Sci. Rep. **11**, 4908 (2021). 33649456 10.1038/s41598-021-84406-3 PMC7921689

[12] E. Moreno, K. Basler, G. Morata, Cells compete for Decapentaplegic survival factor to prevent apoptosis in *Drosophila* wing development. Nature **416**, 755–759 (2002). 11961558 10.1038/416755a

[13] J. P. Vincent, G. Kolahgar, M. Gagliardi, E. Piddini, Steep differences in wingless signaling trigger Myc-independent competitive cell interactions. Dev. Cell **21**, 366–374 (2011). 21839923 10.1016/j.devcel.2011.06.021 PMC3209557

[14] C. M. Paluskievicz , T regulatory cells and priming the suppressive tumor microenvironment. Front. Immunol. **10**, 2453 (2019). 31681327 10.3389/fimmu.2019.02453 PMC6803384

[15] C. H. Chang , Metabolic competition in the Tumor microenvironment is a driver of Cancer progression. Cell **162**, 1229–1241 (2015). 26321679 10.1016/j.cell.2015.08.016 PMC4864363

[16] G. Morata, P. Ripoll, Minutes: Mutants of *Drosophila* autonomously affecting cell division rate. Dev. Biol. **42**, 211–221 (1975). 1116643 10.1016/0012-1606(75)90330-9

[17] A. B. Rodrigues , Activated STAT regulates growth and induces competitive interactions independently of Myc, Yorkie, Wingless and ribosome biogenesis. Development **139**, 4051–4061 (2012). 22992954 10.1242/dev.076760 PMC3472591

[18] P. Simpson, Parameters of cell competition in the compartments of the wing disc of *Drosophila*. Dev. Biol. **69**, 182–193 (1979). 446891 10.1016/0012-1606(79)90284-7

[19] P. Simpson, G. Morata, Differential mitotic rates and patterns of growth in compartments in the *Drosophila* wing. Dev. Biol. **85**, 299–308 (1981). 7262460 10.1016/0012-1606(81)90261-x

[20] L. A. Johnston, D. A. Prober, B. A. Edgar, R. N. Eisenman, P. Gallant, *Drosophila* MYC regulates cellular growth during development. Cell **98**, 779–790 (1999). 10499795 10.1016/s0092-8674(00)81512-3 PMC10176494

[21] N. E. Baker, Mechanisms of cell competition emerging from *Drosophila* studies. Curr. Opin. Cell Biol. **48**, 40–46 (2017). 28600967 10.1016/j.ceb.2017.05.002 PMC5591771

[22] C. C. Soares, A. Rizzo, M. F. Maresma, P. Meier, Autocrine glutamate signaling drives cell competition in *Drosophila*. Dev. Cell **59**, 2974–2989.e5 (2024). 39047739 10.1016/j.devcel.2024.06.022

[23] A. Krotenberg Garcia , Cell competition promotes metastatic intestinal cancer through a multistage process. iScience **27**, 109718 (2024). 38706869 10.1016/j.isci.2024.109718 PMC11068562

[24] E. R. Oliver, T. L. Saunders, S. A. Tarlé, T. Glaser, Ribosomal protein L24 defect in Belly spot and tail (Bst), a mouse Minute. Development **131**, 3907–3920 (2004). 15289434 10.1242/dev.01268 PMC2262800

[25] M. Oertel, A. Menthena, M. D. Dabeva, D. A. Shafritz, Cell competition leads to a high level of normal liver reconstitution by transplanted fetal liver stem/progenitor cells. Gastroenterology **130**, 507–520 (2006). 16472603 10.1053/j.gastro.2005.10.049

[26] C. Clavería, G. Giovinazzo, R. Sierra, M. Torres, Myc-driven endogenous cell competition in the early mammalian embryo. Nature **500**, 39–44 (2013). 23842495 10.1038/nature12389

[27] M. Sancho , Competitive interactions eliminate unfit embryonic stem cells at the onset of differentiation. Dev. Cell **26**, 19–30 (2013). 23867226 10.1016/j.devcel.2013.06.012 PMC3714589

[28] S. J. Ellis , Distinct modes of cell competition shape mammalian tissue morphogenesis. Nature **569**, 497–502 (2019). 31092920 10.1038/s41586-019-1199-y PMC6638572

[29] G. Morata, Cell competition: A historical perspective. Dev. Biol. **476**, 33–40 (2021). 33775694 10.1016/j.ydbio.2021.02.012

[30] M. Ziosi , dMyc functions downstream of yorkie to promote the supercompetitive behavior of hippo pathway mutant cells. PLoS Genet. **6**, e1001140 (2010). 20885789 10.1371/journal.pgen.1001140 PMC2944792

[31] F. D. Karim, G. M. Rubin, Ectopic expression of activated Ras1 induces hyperplastic growth and increased cell death in *Drosophila* imaginal tissues. Development **125**, 1–9 (1998). 9389658 10.1242/dev.125.1.1

[32] R. M. Neto-Silva, S. De Beco, L. A. Johnston, Evidence for a growth-stabilizing regulatory feedback mechanism between Myc and Yorkie, the *Drosophila* homolog of yap. Dev. Cell **19**, 507–520 (2010). 20951343 10.1016/j.devcel.2010.09.009 PMC2965774

[33] D. M. Tyler, W. Li, N. Zhuo, B. Pellock, N. E. Baker, Genes affecting cell competition in *Drosophila*. Genetics **175**, 643–657 (2007). 17110495 10.1534/genetics.106.061929 PMC1800612

[34] M. Amoyel, B. D. Simons, E. A. Bach, Neutral competition of stem cells is skewed by proliferative changes downstream of Hh and Hpo. *EMBO J.* **33**, 2295–2313 (2014). 10.15252/embj.201387500 PMC4253521 25092766

[35] L. Vermeulen , Defining stem cell dynamics in models of intestinal Tumor initiation. Science **342**, 995–998 (2013). 24264992 10.1126/science.1243148

[36] H. J. Snippert, A. G. Schepers, J. H. Van Es, B. D. Simons, H. Clevers, Biased competition between Lgr5 intestinal stem cells driven by oncogenic mutation induces clonal expansion. EMBO Rep. **15**, 62–69 (2014). 24355609 10.1002/embr.201337799 PMC3983678

[37] C. L. G. J. Scheele , Identity and dynamics of mammary stem cells during branching morphogenesis. Nature **542**, 313–317 (2017). 28135720 10.1038/nature21046 PMC6097610

[38] W. Kim, R. Jain, Picking winners and losers: Cell competition in tissue development and homeostasis. Trends Genet. **36**, 490–498 (2020). 32418713 10.1016/j.tig.2020.04.003 PMC7299791

[39] A. Csikász-Nagy , Cooperation and competition in the dynamics of tissue architecture during homeostasis and tumorigenesis. Semin. Cancer Biol. **23**, 293–298 (2013). 23751796 10.1016/j.semcancer.2013.05.009

[40] T. Cumming, R. Levayer, Toward a predictive understanding of epithelial cell death. Semin. Cell Dev. Biol. **156**, 44–57 (2024). 37400292 10.1016/j.semcdb.2023.06.008

[41] R. MacArthur, Species packing and competitive equilibrium for many species. Theor. Popul. Biol. **1**, 1–11 (1970). 5527624 10.1016/0040-5809(70)90039-0

[42] P. Chesson, Macarthur’s consumer-resource model. Theor. Popul. Biol. **37**, 26–38 (1990).

[43] D. Tilman, Resources: A graphical-mechanistic approach to competition and predation. Am. Nat. **116**, 362–393 (1980).

[44] P. J. Wangersky, Lotka-Volterra population models. Annu. Rev. Ecol. Syst. **9**, 189–218 (1978).

[45] R. Cherniha, V. Davydovych, Construction and application of exact solutions of the diffusive Lotka-Volterra system: A review and new results. Commun. Nonlinear Sci. Numer. Simul. **113**, 106579 (2022).

[46] O. Malcai, O. Biham, P. Richmond, S. Solomon, Theoretical analysis and simulations of the generalized Lotka-Volterra model. Phys. Rev. E **66**, 031102 (2002). 10.1103/PhysRevE.66.031102 12366094

[47] R. Chignola, A. D. Fabbro, C. D. Pellegrina, E. Milotti, Ab initio phenomenological simulation of the growth of large tumor cell populations. Phys. Biol. **4**, 114 (2007). 17664656 10.1088/1478-3975/4/2/005

[48] R. de la Cruz, P. Guerrero, J. Calvo, T. Alarcón, Coarse-graining and hybrid methods for efficient simulation of stochastic multi-scale models of tumour growth. J. Comput. Phys. **350**, 974–991 (2017). 29200499 10.1016/j.jcp.2017.09.019 PMC5656096

[49] P. Rdl Cruz, F Spill Guerrero, T. Alarcón, Stochastic multi-scale models of competition within heterogeneous cellular populations: Simulation methods and mean-field analysis. J. Theor. Biol. **407**, 161–183 (2016). 27457092 10.1016/j.jtbi.2016.07.028 PMC5016039

[50] A. V. Ponce Bobadilla, T. Carraro, H. M. Byrne, P. K. Maini, T. Alarcón, Age structure can account for delayed logistic proliferation of scratch assays. Bull. Math. Biol. **81**, 2706–2724 (2019). 31201661 10.1007/s11538-019-00625-w

[51] W. J. Ewens, Mathematical Population Genetics, Interdisciplinary Applied Mathematics (Springer, New York, NY, 2004), vol. 27.

[52] M. A. Nowak, Evolutionary Dynamics: Exploring the Equations of Life (Harvard University Press, 2006).

[53] P. A. P. Moran, Random processes in genetics. Math. Proc. Camb. Philos. Soc. **54**, 60–71 (1958).

[54] S. Wright, Evolution in mendelian populations. Genetics **16**, 97–159 (1931). 17246615 10.1093/genetics/16.2.97 PMC1201091

[55] R. A. Fisher, The Genetical Theory Of Natural Selection (At The Clarendon Press, 1930).

[56] T. Buder, A. Deutsch, B. Klink, A. Voss-Böhme, Patterns of tumor progression predict small and tissue-specific tumor-originating niches. Front. Oncol. **8**, 668 (2019). 30687642 10.3389/fonc.2018.00668 PMC6335293

[57] E. Lieberman, C. Hauert, M. A. Nowak, Evolutionary dynamics on graphs. Nature **433**, 312–316 (2005). 15662424 10.1038/nature03204

[58] J. M. Smith, Evolutionary game theory. Phys. D Nonlinear Phenom. **22**, 43–49 (1986).

[59] G. Szabó, G. Fáth, Evolutionary games on graphs. Phys. Rep. **446**, 97–216 (2007).

[60] M. Akiyama, T. Sushida, S. Ishida, H. Haga, Mathematical model of collective cell migrations based on cell polarity. Dev. Growth Differ. **59**, 471–490 (2017). 28714585 10.1111/dgd.12381

[61] Z. Kirchner, A. Geohagan, A. Truszkowska, A Vicsek-type model of confined cancer cells with variable clustering affinities. Integr. Biol. **16**, zyae005 (2024). 10.1093/intbio/zyae005 38402577

[62] B. A. Camley, J. Zimmermann, H. Levine, W. J. Rappel, Collective signal processing in cluster chemotaxis: Roles of adaptation, amplification, and co-attraction in collective guidance. PLoS Comput. Biol. **12**, e1005008 (2016). 27367541 10.1371/journal.pcbi.1005008 PMC4930173

[63] B. Smeets , Emergent structures and dynamics of cell colonies by contact inhibition of locomotion. Proc. Natl. Acad. Sci. U.S.A. **113**, 14621–14626 (2016). 27930287 10.1073/pnas.1521151113 PMC5187738

[64] B. Szabó , Phase transition in the collective migration of tissue cells: Experiment and model. Phys. Rev. E **74**, 061908 (2006). 10.1103/PhysRevE.74.061908 17280097

[65] J. Li, S. K. Schnyder, M. S. Turner, R. Yamamoto, Competition between cell types under cell cycle regulation with apoptosis. Phys. Rev. Res. **4**, 033156 (2022).

[66] D. B. Staple , Mechanics and remodelling of cell packings in epithelia. Eur. Phys. J. E Soft Matter **33**, 117–127 (2010). 21082210 10.1140/epje/i2010-10677-0

[67] S. Alt, P. Ganguly, G. Salbreux, Vertex models: From cell mechanics to tissue morphogenesis. Philos. Trans. R. Soc. Lond. B Biol. Sci. **372**, 20150520 (2017). 28348254 10.1098/rstb.2015.0520 PMC5379026

[68] Z. Lange, F. Matthäus, M. Qiu, Vertex models capturing subcellular scales in epithelial tissues. PLoS Comput. Biol. **21**, e1012993 (2025). 40397938 10.1371/journal.pcbi.1012993 PMC12094738

[69] D. Bi, J. H. Lopez, J. M. Schwarz, M. L. Manning, A density-independent rigidity transition in biological tissues. *Nat. Phys.* **11**, 1074–1079 (2015).

[70] D. L. Barton, S. Henkes, C. J. Weijer, R. Sknepnek, Active Vertex Model for cell-resolution description of epithelial tissue mechanics. PLoS Comput. Biol. **13**, e1005569 (2017). 28665934 10.1371/journal.pcbi.1005569 PMC5493290

[71] D. Bi, X. Yang, M. C. Marchetti, M. L. Manning, Motility-driven glass and jamming transitions in biological tissues. Phys. Rev. X **6**, 021011 (2016). 28966874 10.1103/PhysRevX.6.021011 PMC5619672

[72] D. M. Sussman, M. Paoluzzi, M. C. Marchetti, M. L. Manning, Anomalous glassy dynamics in simple models of dense biological tissue. Europhys. Lett. **121**, 36001 (2018).

[73] F. Giavazzi , Flocking transitions in confluent tissues. Soft Matter **14**, 3471–3477 (2018). 29693694 10.1039/c8sm00126j PMC5995478

[74] J. Glazier, F. Graner, Simulation of the differential adhesion driven rearrangement of biological cells. Phys. Rev. E **47**, 2128–2154 (1993). 10.1103/physreve.47.2128 9960234

[75] P. Hogeweg, Evolving mechanisms of morphogenesis: On the interplay between differential adhesion and cell differentiation. J. Theor. Biol. **203**, 317–333 (2000). 10736211 10.1006/jtbi.2000.1087

[76] S. E. M. Boas, Y. Jiang, R. M. H. Merks, S. A. Prokopiou, E. G. Rens, “Cellular Potts model: Applications to vasculogenesis and angiogenesis” in *Probabilistic Cellular Automata: Theory, Applications and Future Perspectives*, P. Y. Louis, F. R. Nardi, Eds. (Springer International Publishing, Cham, 2018), pp. 279–310.

[77] A. Szabó, R. M. H. Merks, Cellular Potts modeling of tumor growth, tumor invasion, and tumor evolution. Front. Oncol. **3**, 87 (2013). 23596570 10.3389/fonc.2013.00087 PMC3627127

[78] H. Nemati, J. d. Graaf, The cellular Potts model on disordered lattices. Soft Matter **20**, 8337–8352 (2024). 39283268 10.1039/d4sm00445k PMC11404401

[79] Q. J. S. Braat , Shape matters: Inferring the motility of confluent cells from static images. Soft Matter **21**, 6504–6515 (2025). 40621735 10.1039/d5sm00222b PMC12235246

[80] M. Nonomura, Study on multicellular systems using a phase field model. PLoS One **7**, e33501 (2012). 22539943 10.1371/journal.pone.0033501 PMC3335162

[81] A. Moure, H. Gomez, Phase-field modeling of individual and collective cell migration. Arch. Comput. Methods Eng. **28**, 311–344 (2021).

[82] S. Monfared, A. Ardaševa, A. Doostmohammadi, Multi-phase-field models of biological tissues. arXiv [Preprint] (2025) https://arxiv.org/abs/2503.05053 (Accessed 2 October 2025).

[83] R. Alert, X. Trepat, Physical models of collective cell migration. Ann. Rev. Condens. Matter Phys. **11**, 77–101 (2020).

[84] Y. Jiang, J. Pjesivac-Grbovic, C. Cantrell, J. P. Freyer, A multiscale model for avascular tumor growth. Biophys. J. **89**, 3884–3894 (2005). 16199495 10.1529/biophysj.105.060640 PMC1366955

[85] L. C. Carpenter, S. Banerjee, Physical traits of supercompetitors in cell competition. bioRxiv [Preprint] (2024). https://biorxiv.org/content/10.1101/2024.10.14.618306v1 (Accessed 2 October 2025). 10.1098/rsif.2025.0638 PMC12503947 41056994

[86] L. Brézin, K. S. Korolev, Mechanically-driven growth and competition in a Voronoi model of tissues. arXiv [Preprint] (2024). https://arxiv.org/abs/2405.07899v1 (Accessed 2 October 2025).

[87] A. Schoenit , Force transmission is a master regulator of mechanical cell competition. Nat. Mater. **24**, 966–976 (2025). 40087537 10.1038/s41563-025-02150-9 PMC12133594

[88] F. S. Alsubaie, Z. Neufeld, Modelling the effect of cell motility on mixing and invasion in epithelial monolayers. J. Biol. Phys. **50**, 291–306 (2024). 39031299 10.1007/s10867-024-09660-8 PMC11490479

[89] D. Gradeci , Cell-scale biophysical determinants of cell competition in epithelia. eLife **10**, e61011 (2021). 34014166 10.7554/eLife.61011 PMC8137148

[90] T. F. Pak, J. Pitt-Francis, R. E. Baker, A mathematical framework for the emergence of winners and losers in cell competition. J. Theor. Biol. **577**, 111666 (2024). 37956955 10.1016/j.jtbi.2023.111666 PMC7618079

[91] M. Tang , Nomogram for predicting occurrence and prognosis of liver metastasis in colorectal cancer: A population-based study. Int. J. Colorectal Dis. **36**, 271–282 (2021). 32965529 10.1007/s00384-020-03722-8

[92] J. Engstrand, H. Nilsson, C. Strömberg, E. Jonas, J. Freedman, Colorectal cancer liver metastases - a population-based study on incidence, management and survival. BMC Cancer **18**, 78 (2018). 29334918 10.1186/s12885-017-3925-x PMC5769309

[93] D. I. Tsilimigras , Liver metastases. Nat. Rev. Dis. Prim. **7**, 1–23 (2021). 33859205 10.1038/s41572-021-00261-6

[94] A. Krotenberg Garcia, J. van Rheenen, S. J. E. Suijkerbuijk, Generation of mixed murine organoids to model cellular interactions. STAR Protoc. **2**, 100997 (2021). 34917977 10.1016/j.xpro.2021.100997 PMC8666359

[95] M. Lamprou, A. Krotenberg Garcia, S. J. E. Suijkerbuijk, Protocol for generating liver metastasis microtissues to decipher cellular interactions between metastatic intestinal cancer and liver tissue. STAR Protoc. **6**, 103575 (2025). 39836518 10.1016/j.xpro.2024.103575 PMC11787675

[96] T. Abe , Visualization of cell cycle in mouse embryos with Fucci2 reporter directed by Rosa26 promoter. Development **140**, 237–246 (2013). 23175634 10.1242/dev.084111

[97] D. Kong , Growth differentiation factor 7 autocrine signaling promotes hepatic progenitor cell expansion in liver fibrosis. Stem Cell Res. Ther. **14**, 288 (2023). 37798809 10.1186/s13287-023-03493-3 PMC10557292

[98] L. Yang , Sonic hedgehog is an autocrine viability factor for myofibroblastic hepatic stellate cells. J. Hepatol. **48**, 98–106 (2008). 18022723 10.1016/j.jhep.2007.07.032 PMC2196213

[99] E. Santoni-Rugiu, P. Jelnes, S. S. Thorgeirsson, H. C. Bisgaard, Progenitor cells in liver regeneration: Molecular responses controlling their activation and expansion. APMIS **113**, 876–902 (2005). 16480456 10.1111/j.1600-0463.2005.apm_386.x

[100] D. S. Salomon, N. Kim, T. Saeki, F. Ciardiello, Transforming growth factor-alpha: An oncodevelopmental growth factor. Cancer Cells **2**, 389–397 (1990). 2088454

[101] G. K. Michalopoulos, Hepatostat: Liver regeneration and normal liver tissue maintenance. Hepatology **65**, 1384–1392 (2017). 27997988 10.1002/hep.28988

[102] A. Sánchez, I. Fabregat, Growth factor- and cytokine-driven pathways governing liver stemness and differentiation. World J. Gastroenterol. **16**, 5148–5161 (2010). 21049549 10.3748/wjg.v16.i41.5148 PMC2975086

[103] Y. Yarden, M. X. Sliwkowski, Untangling the ErbB signalling network. Nat. Rev. Mol. Cell Biol. **2**, 127–137 (2001). 11252954 10.1038/35052073

[104] A. M. Sizeland, A. W. Burgess, Anti-sense transforming growth factor alpha oligonucleotides inhibit autocrine stimulated proliferation of a colon carcinoma cell line. Mol. Biol. Cell **3**, 1235–1243 (1992). 1457828 10.1091/mbc.3.11.1235 PMC275690

[105] T. Lamonerie, C. Lavialle, H. Haddada, O. Brison, IGF-2 autocrine stimulation in tumorigenic clones of a human colon-carcinoma cell line. Int. J. Cancer **61**, 587–592 (1995). 7759165 10.1002/ijc.2910610425

[106] M. V. Blagosklonny, A. B. Pardee, The restriction point of the cell cycle. Cell Cycle **1**, 102–109 (2002). 12429916

[107] M. D. Planas-Silva, R. A. Weinberg, The restriction point and control of cell proliferation. Curr. Opin. Cell Biol. **9**, 768–772 (1997). 9425340 10.1016/s0955-0674(97)80076-2

[108] A. B. Pardee, A restriction point for control of normal animal cell proliferation. Proc. Natl. Acad. Sci. U.S.A. **71**, 1286–1290 (1974). 4524638 10.1073/pnas.71.4.1286 PMC388211

[109] A. Johnson, J. M. Skotheim, Start and the restriction point. Curr. Opin. Cell Biol. **25**, 717–723 (2013). 23916770 10.1016/j.ceb.2013.07.010 PMC3836907

[110] A. B. Pardee, R. Dubrow, J. L. Hamlin, R. F. Kletzien, Animal cell cycle. Annu. Rev. Biochem. **47**, 715–750 (1978). 354504 10.1146/annurev.bi.47.070178.003435

[111] S. A. Ezhevsky , Hypo-phosphorylation of the retinoblastoma protein (pRb) by cyclin D:Cdk4/6 complexes results in active-pRb. Proc. Natl. Acad. Sci. U.S.A. **94**, 10699–10704 (1997). 9380698 10.1073/pnas.94.20.10699 PMC23451

[112] A. Zetterberg, O. Larsson, K. G. Wiman, What is the restriction point? Curr. Opin. Cell Biol. **7**, 835–842 (1995). 8608014 10.1016/0955-0674(95)80067-0

[113] J. P. Spano , Epidermal growth factor receptor signaling in colorectal cancer: Preclinical data and therapeutic perspectives. Ann. Oncol. **16**, 189–194 (2005). 15668269 10.1093/annonc/mdi057

[114] Z. Wang, Regulation of cell cycle progression by growth factor-induced cell signaling. Cells **10**, 3327 (2021). 34943835 10.3390/cells10123327 PMC8699227

[115] D. A. Foster, P. Yellen, L. Xu, M. Saqcena, Regulation of G1 cell cycle progression: Distinguishing the restriction point from a nutrient-sensing cell growth checkpoint(s). Genes Cancer **1**, 1124–1131 (2010). 21779436 10.1177/1947601910392989 PMC3092273

[116] E. Hulleman, J. Boonstra, Regulation of G1 phase progression by growth factors and the extracellular matrix. CMLS **58**, 80–93 (2001). 11229819 10.1007/PL00000780 PMC11146479

[117] A. Lukaszewicz, P. Savatier, V. Cortay, H. Kennedy, C. Dehay, Contrasting effects of basic fibroblast growth factor and neurotrophin 3 on cell cycle kinetics of mouse cortical stem cells. J. Neurosci. Nurs. **22**, 6610–6622 (2002). 10.1523/JNEUROSCI.22-15-06610.2002 PMC2001296 12151540

[118] N. Molinie , Cortical branched actin determines cell cycle progression. Cell Res. **29**, 432–445 (2019). 30971746 10.1038/s41422-019-0160-9 PMC6796858

[119] R. D. Hodge, A. J. D’Ercole, J. R. O’Kusky, Insulin-like growth factor-i accelerates the cell cycle by decreasing g1phase length and increases cell cycle reentry in the embryonic cerebral cortex. J. Neurosci. Nurs. **24**, 10201–10210 (2004). 10.1523/JNEUROSCI.3246-04.2004 PMC6730172 15537892

[120] P. Icard, L. Fournel, Z. Wu, M. Alifano, H. Lincet, Interconnection between metabolism and cell cycle in cancer. Trends Biochem. Sci. **44**, 490–501 (2019). 30655165 10.1016/j.tibs.2018.12.007

[121] P. Dong, C. Zhang, B. T. Parker, L. You, B. Mathey-Prevot, Cyclin D/CDK4/6 activity controls G1 length in mammalian cells. PLoS One **13**, e0185637 (2018). 29309421 10.1371/journal.pone.0185637 PMC5757913

[122] P. Jain , Cell-state transitions and density-dependent interactions together explain the dynamics of spontaneous epithelial-mesenchymal heterogeneity. iScience **27**, 110310 (2024). 39055927 10.1016/j.isci.2024.110310 PMC11269952

[123] M. J. Simpson, A. P. Browning, D. J. Warne, O. J. Maclaren, R. E. Baker, Parameter identifiability and model selection for sigmoid population growth models. J. Theor. Biol. **535**, 110998 (2022). 34973274 10.1016/j.jtbi.2021.110998

[124] G. Bellu, M. P. Saccomani, S. Audoly, L. D’Angiò, Daisy: A new software tool to test global identifiability of biological and physiological systems. Comput. Methods Programs Biomed. **88**, 52–61 (2007). 17707944 10.1016/j.cmpb.2007.07.002 PMC2888537

[125] M. J. Simpson, M. J. Plank, Inference and prediction for stochastic models of biological populations undergoing migration and proliferation. J. R. Soc. Interface **22**, 20250536 (2025). 41151767 10.1098/rsif.2025.0536 PMC12567133

[126] A. Raue , Structural and practical identifiability analysis of partially observed dynamical models by exploiting the profile likelihood. Bioinformatics **25**, 1923–1929 (2009). 19505944 10.1093/bioinformatics/btp358

[127] B. Boward, T. Wu, S. Dalton, Concise review: Control of cell fate through cell cycle and pluripotency networks. Stem Cells **34**, 1427–1436 (2016). 26889666 10.1002/stem.2345 PMC5201256

[128] H. M. Blank, M. Callahan, I. P. E. Pistikopoulos, A. O. Polymenis, M. Polymenis, Scaling of G1 duration with population doubling time by a cyclin in saccharomyces cerevisiae. Genetics **210**, 895–906 (2018). 30150288 10.1534/genetics.118.301507 PMC6218239

[129] A. E. DeWitt, J. Y. Dong, H. S. Wiley, D. A. Lauffenburger, Quantitative analysis of the EGF receptor autocrine system reveals cryptic regulation of cell response by ligand capture. J. Cell Sci. **114**, 2301–2313 (2001). 11493669 10.1242/jcs.114.12.2301

[130] E. J. Joslin, L. K. Opresko, A. Wells, H. S. Wiley, D. A. Lauffenburger, EGF-receptor-mediated mammary epithelial cell migration is driven by sustained ERK signaling from autocrine stimulation. J. Cell Sci. **120**, 3688–3699 (2007). 17895366 10.1242/jcs.010488

[131] S. Dalton, Linking the cell cycle to cell fate decisions. Trends Cell Biol. **25**, 592–600 (2015). 26410405 10.1016/j.tcb.2015.07.007 PMC4584407

[132] A. Picot, T. Monnin, N. Loeuille, From apparent competition to facilitation: Impacts of consumer niche construction on the coexistence and stability of consumer-resource communities. Funct. Ecol. **33**, 1746–1757 (2019).

[133] W. Cui, R. Marsland, P. Mehta, Diverse communities behave like typical random ecosystems. Phys. Rev. E **104**, 034416 (2021). 34654170 10.1103/PhysRevE.104.034416 PMC9005152

[134] D. Walker, S. Wood, J. Southgate, M. Holcombe, R. Smallwood, An integrated agent-mathematical model of the effect of intercellular signalling via the epidermal growth factor receptor on cell proliferation. J. Theor. Biol. **242**, 774–789 (2006). 16765384 10.1016/j.jtbi.2006.04.020

[135] H. Nemati, Data from “Cell competition driven by secreted ligands: Modeling liver metastasis of colorectal cancer.” Yoda. 10.24416/uu01-da6tug. Deposited 3 August 2026. PMC13535163 42658767

