# Supplementary material for: Cell competition driven by secreted ligands: Modeling liver metastasis of colorectal cancer
Source: Proc Natl Acad Sci U S A. 2026 Aug 27;123(35):e2531078123. doi: 10.1073/pnas.2531078123 (PMC13535163; doi:10.1073/pnas.2531078123)
Supplement: Supplementary file 1 [file pnas.2531078123.sapp.pdf]

PNAS

Supporting Information for

Cell Competition Driven by Secreted Ligands: Modeling Liver Metastasis of Colorectal Cancer

Hossein Nemati, Saskia Jacoba Elisabeth Suijkerbuijk and Joost de Graaf

Hossein Nemati.
E-mail: h.nemati@uu.nl

This PDF file includes:

- Supporting text
- Figs. S1 to S6
- Table S1
- Legend for Dataset S1
- SI References

Other supporting materials for this manuscript include the following:

- Dataset S1

Supporting Information Text

Derivation of solutions for pure populations

In this section, we derive the analytical solutions to the dimensionless forms of Eqs. 2, 5 and 6 for pure wild-type and pure cancer organoids. For convenience, we present the dimensionless form of Eqs. 1–6 of the manuscript(for $x \in \{\mathrm{W}, \mathrm{C}\}$):

$$\tilde{v}^{x}_{\mathrm{G1}}(\tilde{t}) \;=\; \tilde{\mu}_x\, \tilde{\alpha}(\tilde{t}) = \tilde{\mu}_x\, \tilde{M}(\tilde{t})/N(\tilde{t}); \tag{1}$$

$$\frac{\partial \rho^x}{\partial \tilde{t}} = -\frac{\partial}{\partial \phi}\left(\rho^x \tilde{v}^x\right) - \tilde{r}^x \rho^x; \tag{2}$$

$$\tilde{v}^{x}_{\mathrm{G1}}(\tilde{t})\, \rho^x(\pi^-, \tilde{t}) = \tilde{v}_{\mathrm{S}}\, \rho^x(\pi^+, \tilde{t}); \tag{3}$$

$$\tilde{v}^{x}_{\mathrm{G1}}(\tilde{t})\, \rho^x(0, \tilde{t}) = 2\, \tilde{v}_{\mathrm{S}}\, \rho^x(2\pi, \tilde{t}); \tag{4}$$

$$\frac{d\tilde{M}}{d\tilde{t}} = \left(\tilde{S} - \tilde{\mu}_{\mathrm{W}}\, \tfrac{\tilde{M}}{N}\right) W(\tilde{t}) \;+\; \left(\tilde{S} - \tilde{\mu}_{\mathrm{C}}\, \tfrac{\tilde{M}}{N}\right) C(\tilde{t}); \tag{5}$$

$$\frac{dW_{\mathrm{D}}}{d\tilde{t}} = \tilde{r}_{\mathrm{D}} \int_0^{\pi} \rho^{\mathrm{W}}(\phi, \tilde{t})\, d\phi. \tag{6}$$

From this point onward, ρ_{G1} denotes $\rho(\phi, \tilde{t})$ for $\phi \in (0, \pi)$, and ρ_{S} denotes $\rho(\phi, \tilde{t})$ for $\phi \in (\pi, 2\pi)$. The letters W and C refer to wild-type and cancer, respectively.

Pure wild-type solution. For pure organoids—comprising either wild-type or cancer cells—we assume that, on average, the organoids begin in a quasi-steady state and remain in that state throughout the experiments. In the quasi-steady state, the distribution of cells in ϕ-space should not change, which means:

$$\frac{\partial}{\partial \tilde{t}}\left(\frac{1}{W(\tilde{t})}\, \rho^{\mathrm{W}}(\phi, \tilde{t})\right) = 0, \tag{7}$$

and

$$\frac{\partial}{\partial \tilde{t}}\left(\frac{W_{\mathrm{D}}(\tilde{t})}{W(\tilde{t})}\right) = 0. \tag{8}$$

Equation 7 requires that the progression speed through the G1 phase is constant, which in turn, requires a constant equilibrium concentration of the growth factor. So, we have:

$$\tilde{v}^{\mathrm{W}}_{\mathrm{G1}} = \tilde{\mu}_{\mathrm{W}} \tilde{\alpha}_{\mathrm{W}}. \tag{9}$$

With this, Eqs. 2, 6, and 5 simplify to:

$$\frac{\partial \rho^{\mathrm{W}}_{\mathrm{G1}}}{\partial \tilde{t}} \;+\; \tilde{\mu}_{\mathrm{W}} \tilde{\alpha}_{\mathrm{W}}\, \frac{\partial \rho^{\mathrm{W}}_{\mathrm{G1}}}{\partial \phi} \;+\; \tilde{r}_{\mathrm{D}}\, \rho^{\mathrm{W}}_{\mathrm{G1}} = 0; \tag{10}$$

$$\frac{\partial \rho^{\mathrm{W}}_{\mathrm{S}}}{\partial \tilde{t}} \;+\; \tilde{v}_{\mathrm{S}}\, \frac{\partial \rho^{\mathrm{W}}_{\mathrm{S}}}{\partial \phi} = 0; \tag{11}$$

$$\frac{dW_{\mathrm{D}}}{d\tilde{t}} = \tilde{r}_{\mathrm{D}}\, W_{\mathrm{G1}}(\tilde{t}); \tag{12}$$

$$\frac{d\,\tilde{M}}{d\tilde{t}} = \left(\tilde{S} \;-\; \tilde{\mu}_{\mathrm{W}} \tilde{\alpha}_{\mathrm{W}}\right) W(\tilde{t}) \;=\; \tilde{S} W(\tilde{t}) \;-\; \tilde{\mu}_{\mathrm{W}} \tilde{M}(\tilde{t}). \tag{13}$$

According to the experiments for the pure wild-type organoids:

$$W(\tilde{t}) = W(0)\, e^{\tilde{t}}, \tag{14}$$

and the differentiating cells:

$$W_{\mathrm{D}}(\tilde{t}) = f_{\mathrm{D}} W(\tilde{t}) = f_{\mathrm{D}}\; W(0)\, e^{\tilde{t}}, \tag{15}$$

where f_{D} is the fraction of differentiating wild-type cells, which approximately equals to 0.2 according to the experiments reported in Ref. 1. Given that $\tilde{M}(\tilde{t}) = \tilde{\alpha}_{\mathrm{W}} W(\tilde{t})$ and by substituting Eq. 14 in Eq. 13, we find the equilibrium concentration for the pure wild-type organoids:

$$\tilde{\alpha}_{\mathrm{W}} = \frac{\tilde{S}}{1 + \tilde{\mu}_{\mathrm{W}}}. \tag{16}$$

We use this equilibrium value for the initial condition of $\tilde{M}$:

$$\tilde{M}(0) = \tilde{\alpha}_{\mathrm{W}}\, W(0). \tag{17}$$

Now we find the density of cells in cell phase. Combining Eqs. 7 and 14 results in:

$$\rho^{\mathrm{W}}_{\mathrm{G1}}(\phi,\tilde{t}) = f_{\mathrm{W}}(\phi)\, e^{\tilde{t}} \quad \text{for} \quad \phi \in (0,\pi)\ ; \quad \text{and} \quad \rho^{\mathrm{W}}_{\mathrm{S}}(\phi,\tilde{t}) = h_{\mathrm{W}}(\phi)\, e^{\tilde{t}} \quad \text{for} \quad \phi \in (\pi, 2\pi). \qquad [18]$$

By substituting these equations into Eqs. 10 and 11, the functions $f_{\mathrm{W}}(\phi)$ and $h_{\mathrm{W}}(\phi)$ are found (up to a constant factor) as

$$f_{\mathrm{W}}(\phi) = f_{\mathrm{W}}(0)\, \exp\!\left(-\frac{1+\tilde{r}_{\mathrm{D}}}{\tilde{\mu}_{\mathrm{W}}\tilde{\alpha}_{\mathrm{W}}}\,\phi\right); \qquad [19]$$

$$h_{\mathrm{W}}(\phi) = h_{\mathrm{W}}(\pi)\, \exp\!\left(-\frac{\phi-\pi}{\tilde{v}_{\mathrm{S}}}\right). \qquad [20]$$

These functions show exponential distributions for the density of cells in ϕ. Consequently, we will have the densities:

$$\rho^{\mathrm{W}}_{\mathrm{G1}}(\phi,\tilde{t}) = \rho^{\mathrm{W}}_{\mathrm{G1}}(0,0)\, \exp\!\left(-\frac{1+\tilde{r}_{\mathrm{D}}}{\tilde{\mu}_{\mathrm{W}}\tilde{\alpha}_{\mathrm{W}}}\,\phi\right)\, e^{\tilde{t}}; \qquad [21]$$

$$\rho^{\mathrm{W}}_{\mathrm{S}}(\phi,\tilde{t}) = \rho^{\mathrm{W}}_{\mathrm{S}}(\pi,0)\, \exp\!\left(-\frac{\phi-\pi}{\tilde{v}_{\mathrm{S}}}\right)\, e^{\tilde{t}}. \qquad [22]$$

By applying the boundary conditions in Eqs. 3 and 4, we have:

$$\rho^{\mathrm{W}}_{\mathrm{S}}(\pi,0) = \rho^{\mathrm{W}}_{\mathrm{G1}}(0,0)\, \frac{\tilde{\mu}_{\mathrm{W}}\tilde{\alpha}_{\mathrm{W}}}{\tilde{v}_{\mathrm{S}}}\, e^{-m\pi}, \qquad [23]$$

and

$$m + \frac{1}{\tilde{v}_{\mathrm{S}}} = \frac{\ln 2}{\pi}, \qquad [24]$$

where for brevity, we have introduced m:

$$m = \frac{1+\tilde{r}_{\mathrm{D}}}{\tilde{\mu}_{\mathrm{W}}\tilde{\alpha}_{\mathrm{W}}} = \frac{1+\tilde{r}_{\mathrm{D}}}{\tilde{\mu}_{\mathrm{W}}\frac{\tilde{S}}{1+\tilde{\mu}_{\mathrm{W}}}}. \qquad [25]$$

The factor $\rho^{\mathrm{W}}_{\mathrm{G1}}(0,0)$ determines the initial population of wild-type cells, because:

$$\int_0^{2\pi} \rho^{\mathrm{W}}(\phi,0)\, d\phi = (1-f_{\mathrm{D}})W(0). \qquad [26]$$

Hence, the expression for $\rho^{\mathrm{W}}_{\mathrm{G1}}(0,0)$ is:

$$\begin{aligned}\rho^{\mathrm{W}}_{\mathrm{G1}}(0,0) &= (1-f_{\mathrm{D}})W(0)\left(\frac{1}{m}(1-e^{-m\pi}) + \tilde{\mu}_{\mathrm{W}}\,\tilde{\alpha}_{\mathrm{W}}\, e^{-m\pi}\,(1-e^{-\pi/\tilde{v}_{\mathrm{S}}})\right)^{-1} \\ &= 2m(1-f_{\mathrm{D}})W(0)/\left(1+\tilde{r}_{\mathrm{D}}\left(e^{\pi/\tilde{v}_{\mathrm{S}}}-1\right)\right).\end{aligned} \qquad [27]$$

Additionally, we force to keep the fraction of differentiating cells equal to $f_{\mathrm{D}} = 0.2$, which is based on the experimental data. Therefore, we find

$$\frac{dW_{\mathrm{D}}}{d\tilde{t}} = f_{\mathrm{D}}\; W(0)\; e^{\tilde{t}} = \tilde{r}_{\mathrm{D}} W_{\mathrm{G1}}(\tilde{t}) = \tilde{r}_{\mathrm{D}} \int_0^{\pi} \rho^{\mathrm{W}}_{\mathrm{G1}}(\phi,\tilde{t})\; d\phi, \qquad [28]$$

which leads to

$$(1+\tilde{r}_{\mathrm{D}})\left(\frac{1-e^{-\pi/\tilde{v}_{\mathrm{S}}}}{e^{m\pi}-1}\right) + 1 - \tilde{r}_{\mathrm{D}}\left(\frac{1-f_{\mathrm{D}}}{f_{\mathrm{D}}}\right) = 0, \qquad [29]$$

where the value of m is as introduced in Eq. 25. Now, by substituting m from Eq. 24 into Eq. 29, we obtain:

$$\tilde{r}_{\mathrm{D}} = f_{\mathrm{D}}/\left(2 - f_{\mathrm{D}} - \exp(\pi/\tilde{v}_{\mathrm{S}})\right). \qquad [30]$$

Consequently, combining Eqs. 24 and 25, leads to:

$$\tilde{\mu}_{\mathrm{W}} = \left(\frac{\tilde{S}}{1+\tilde{r}_{\mathrm{D}}}\left(\frac{\ln 2}{\pi} - \frac{1}{\tilde{v}_{\mathrm{S}}}\right) - 1\right)^{-1} = 1/\left(\tilde{S}m/\left(1+\tilde{r}_{\mathrm{D}}\right) - 1\right). \qquad [31]$$

In summary, the quasi-steady state solution for the pure wild-type cells is:

Pure wild-type solution

$$\rho^{\mathrm{W}}_{\mathrm{G1}}(\phi,\tilde{t}) = \rho^{\mathrm{W}}_{\mathrm{G1}}(0,0)\, \exp\!\left(-\frac{1+\tilde{r}_{\mathrm{D}}}{\tilde{\mu}_{\mathrm{W}}\tilde{\alpha}_{\mathrm{W}}}\,\phi\right)\, e^{\tilde{t}}; \qquad [32]$$

$$\rho^{\mathrm{W}}_{\mathrm{S}}(\phi,\tilde{t}) = \rho^{\mathrm{W}}_{\mathrm{G1}}(0,0)\, e^{-m\pi}\, \frac{\tilde{\mu}_{\mathrm{W}}\tilde{\alpha}_{\mathrm{W}}}{\tilde{v}_{\mathrm{S}}}\, \exp\!\left(-\frac{\phi-\pi}{\tilde{v}_{\mathrm{S}}}\right)\, e^{\tilde{t}}; \qquad [33]$$

$$W_{\mathrm{D}}(\tilde{t}) = f_{\mathrm{D}}\; W(0)\; e^{\tilde{t}}; \qquad [34]$$

$$\tilde{M}(\tilde{t}) = \tilde{\alpha}_{\mathrm{W}}\; W(0)\; e^{\tilde{t}}. \qquad [35]$$

Pure cancer solution. For pure cancer organoids, we adopt an approach analogous to that used for pure wild-type population. Under quasi-steady-state conditions:

$$\frac{\partial}{\partial \tilde{t}}\left(\frac{1}{C(\tilde{t})}\,\rho^{\mathrm{C}}(\phi,\tilde{t})\right) = 0. \qquad [36]$$

Thus, the progression speed of cells in the G1 phase, and the concentration of the growth factor remain constant. So, we have:

$$\tilde{v}^{\mathrm{C}}_{\mathrm{G1}} = \tilde{\mu}_{\mathrm{C}}\tilde{\alpha}_{\mathrm{C}}, \qquad [37]$$

where $\tilde{\alpha}_{\mathrm{C}}$ is the equilibrium concentration of growth factor in pure cancer organoids. Therefore, the Eqs. 2 and 5 read:

$$\frac{\partial \rho^{\mathrm{C}}_{\mathrm{G1}}}{\partial \tilde{t}} \;+\; \tilde{\mu}_{\mathrm{C}}\tilde{\alpha}_{\mathrm{C}}\,\frac{\partial \rho^{\mathrm{C}}_{\mathrm{G1}}}{\partial \phi} = 0; \qquad [38]$$

$$\frac{\partial \rho^{\mathrm{C}}_{\mathrm{S}}}{\partial \tilde{t}} \;+\; \tilde{v}_{\mathrm{S}}\,\frac{\partial \rho^{\mathrm{C}}_{\mathrm{S}}}{\partial \phi} = 0; \qquad [39]$$

$$\frac{d\tilde{M}}{d\tilde{t}} \;=\; \left(\tilde{S} \;-\; \tilde{\mu}_{\mathrm{C}}\tilde{\alpha}_{\mathrm{C}}\right)C(\tilde{t}) \;=\; \tilde{S}C(\tilde{t}) \;-\; \tilde{\mu}_{\mathrm{C}}\tilde{M}(\tilde{t}). \qquad [40]$$

From the experiments:

$$C(\tilde{t}) = C(0)\,e^{\beta_{\mathrm{C}}\frac{\tilde{t}}{\beta_{\mathrm{W}}}} = C(0)\,e^{\gamma\tilde{t}}, \qquad [41]$$

where we denote the ratio $\beta_{\mathrm{C}}/\beta_{\mathrm{W}}$ by γ. By substituting $\tilde{M}(\tilde{t}) = \tilde{\alpha}_{\mathrm{C}}C(\tilde{t})$ and Eq. 41 in Eq. 40, we can find $\tilde{\alpha}_{\mathrm{C}}$:

$$\tilde{\alpha}_{\mathrm{C}} = \frac{\tilde{S}}{\gamma + \tilde{\mu}_{\mathrm{C}}}. \qquad [42]$$

For the initial condition:

$$\tilde{M}(0) = \tilde{\alpha}_{\mathrm{C}}\,C(0). \qquad [43]$$

Combining Eq. 36 with Eq. 41 results in:

$$\rho^{\mathrm{C}}_{\mathrm{G1}}(\phi,\tilde{t}) = f_{\mathrm{C}}(\phi)\,e^{\gamma\tilde{t}} \quad \text{for} \quad \phi \in (0,\pi)\,; \qquad [44]$$

$$\rho^{\mathrm{C}}_{\mathrm{S}}(\phi,\tilde{t}) = h_{\mathrm{C}}(\phi)\,e^{\gamma\tilde{t}}, \quad \text{for} \quad \phi \in (\pi,2\pi). \qquad [45]$$

By substituting these equations into Eqs. 38 and 39 we have the following.

$$f_{\mathrm{C}}(\phi) = f_{\mathrm{C}}(0)\,\exp\!\left(-\frac{\gamma}{\tilde{\mu}_{\mathrm{C}}\tilde{\alpha}_{\mathrm{C}}}\,\phi\right); \qquad [46]$$

$$h_{\mathrm{C}}(\phi) = h_{\mathrm{C}}(\pi)\,\exp\!\left(-\frac{\gamma}{\tilde{v}_{\mathrm{S}}}(\phi-\pi)\right). \qquad [47]$$

Hence, for the densities are found:

$$\rho^{\mathrm{C}}_{\mathrm{G1}}(\phi,\tilde{t}) = \rho^{\mathrm{C}}_{\mathrm{G1}}(0,0)\,\exp\!\left(-\frac{\gamma}{\tilde{\mu}_{\mathrm{C}}\tilde{\alpha}_{\mathrm{C}}}\,\phi\right)\,e^{\gamma\tilde{t}}; \qquad [48]$$

$$\rho^{\mathrm{C}}_{\mathrm{S}}(\phi,\tilde{t}) = \rho^{\mathrm{C}}_{\mathrm{S}}(\pi,0)\,\exp\!\left(-\frac{\gamma}{\tilde{v}_{\mathrm{S}}}(\phi-\pi)\right)\,e^{\gamma\tilde{t}}. \qquad [49]$$

From the boundary conditions for cancer cells in Eqs. 3 and 4, we derive:

$$\rho^{\mathrm{C}}_{\mathrm{S}}(\pi,0) = \rho^{\mathrm{C}}_{\mathrm{G1}}(0,0)\,e^{-q\pi}\,\frac{\tilde{\mu}_{\mathrm{C}}\tilde{\alpha}_{\mathrm{C}}}{\tilde{v}_{\mathrm{S}}}, \qquad [50]$$

and

$$q + \frac{\gamma}{\tilde{v}_{\mathrm{S}}} = \frac{\ln 2}{\pi}, \qquad [51]$$

where q is:

$$q = \frac{\gamma}{\tilde{\mu}_{\mathrm{C}}\tilde{\alpha}_{\mathrm{C}}} = \frac{\gamma}{\tilde{\mu}_{\mathrm{C}}\,\frac{\tilde{S}}{\gamma+\tilde{\mu}_{\mathrm{C}}}}. \qquad [52]$$

The factor $\rho^{\mathrm{C}}_{\mathrm{G1}}(0,0)$ is determined by the initial number of cells:

$$\int_0^{2\pi} \rho^{\mathrm{C}}(\phi,0)\;d\phi = C(0), \qquad [53]$$

so we find:

$$\rho^{\mathrm{C}}_{\mathrm{G1}}(0,0) = C(0)\left(\frac{1}{q}(1-e^{-q\pi}) + \frac{\tilde{\mu}_{\mathrm{C}}\,\tilde{\alpha}_{\mathrm{C}}}{\gamma}\,e^{-q\pi}\,(1-e^{-\gamma\pi/\tilde{v}_{\mathrm{S}}})\right)^{-1} = 2qC(0), \qquad [54]$$

and

$$\rho_{\mathrm{S}}^{\mathrm{C}}(\pi^{+},0) = C(0)\ (\gamma/\tilde{v}_{\mathrm{S}})\ e^{\gamma\pi/\tilde{v}_{\mathrm{S}}}. \qquad [55]$$

Finally, combining Eqs. 52 and 51 leads to:

$$\tilde{\mu}_{\mathrm{C}} = \gamma \left(\frac{\tilde{S}}{\gamma} \left(\frac{\ln 2}{\pi} - \frac{\gamma}{\tilde{v}_{\mathrm{S}}} \right) - 1 \right)^{-1} = \gamma^2 / \left(\tilde{S}q - \gamma \right). \qquad [56]$$

Hence, the complete solution for pure cancer organoids is as follows.

Pure cancer solution

$$\rho_{\mathrm{G1}}^{\mathrm{C}}(\phi,\tilde{t}) = \rho_{\mathrm{G1}}^{\mathrm{C}}(0,0)\ \exp\left(-\frac{\gamma}{\tilde{\mu}_{\mathrm{C}}\tilde{\alpha}_{\mathrm{C}}}\ \phi \right)\ e^{\gamma\tilde{t}}; \qquad [57]$$

$$\rho_{\mathrm{S}}^{\mathrm{C}}(\phi,\tilde{t}) = \rho_{\mathrm{G1}}^{\mathrm{C}}(0,0)\, e^{-q\pi}\ \frac{\tilde{\mu}_{\mathrm{C}}\tilde{\alpha}_{\mathrm{C}}}{\tilde{v}_{\mathrm{S}}}\ \exp\left(-\frac{\gamma}{\tilde{v}_{\mathrm{S}}}(\phi - \pi) \right)\ e^{\gamma\tilde{t}}; \qquad [58]$$

$$\tilde{M}(\tilde{t}) = \tilde{\alpha}_{\mathrm{C}}\ C(0)\ e^{\gamma\tilde{t}}. \qquad [59]$$

The distributions of the initial number of cells

As a input of the model, we needed samples for initial number of cells in mixed organoids. In order to obtain these statistics, we used the initial numbers of cells in the experiments. We first fitted distributions to the experimental data on the initial number of cells. These fittings as well as the data (taken from Ref. 1) are shown in Fig. S1. For cancer cells in their pure conditions we used Gamma distribution, shown in Eq. 60, while for the other three cases we used exponential distribution, shown in Eq. 61.

$$f(n) = \frac{(n - n_0)^{\alpha-1}}{\Gamma(\alpha)\,\theta^{\alpha}}\ \exp\left(-\frac{n - n_0}{\theta}\right) \quad \text{for} \quad n > n_0; \tag{60}$$

$$f(n) = \frac{1}{\theta}\ \exp\left(-\frac{n - n_0}{\theta}\right) \quad \text{for} \quad n > n_0. \tag{61}$$

For the fittings, we used SciPy package in Python, that uses maximum likelihood estimation (MLE) to find the parameters of the fitted functions. The parameters of the fitted functions are presented in Table S1. Once we found the fitted distributions, we drew random samples with these distributions to use in the initial condition of the model. It is worth noting that for pure organoids, the initial number of cells was always set to one. Hence, only the distributions shown in panels (c) and (d) of Fig. S1 were used. The fittings for pure organoids are included for completeness.

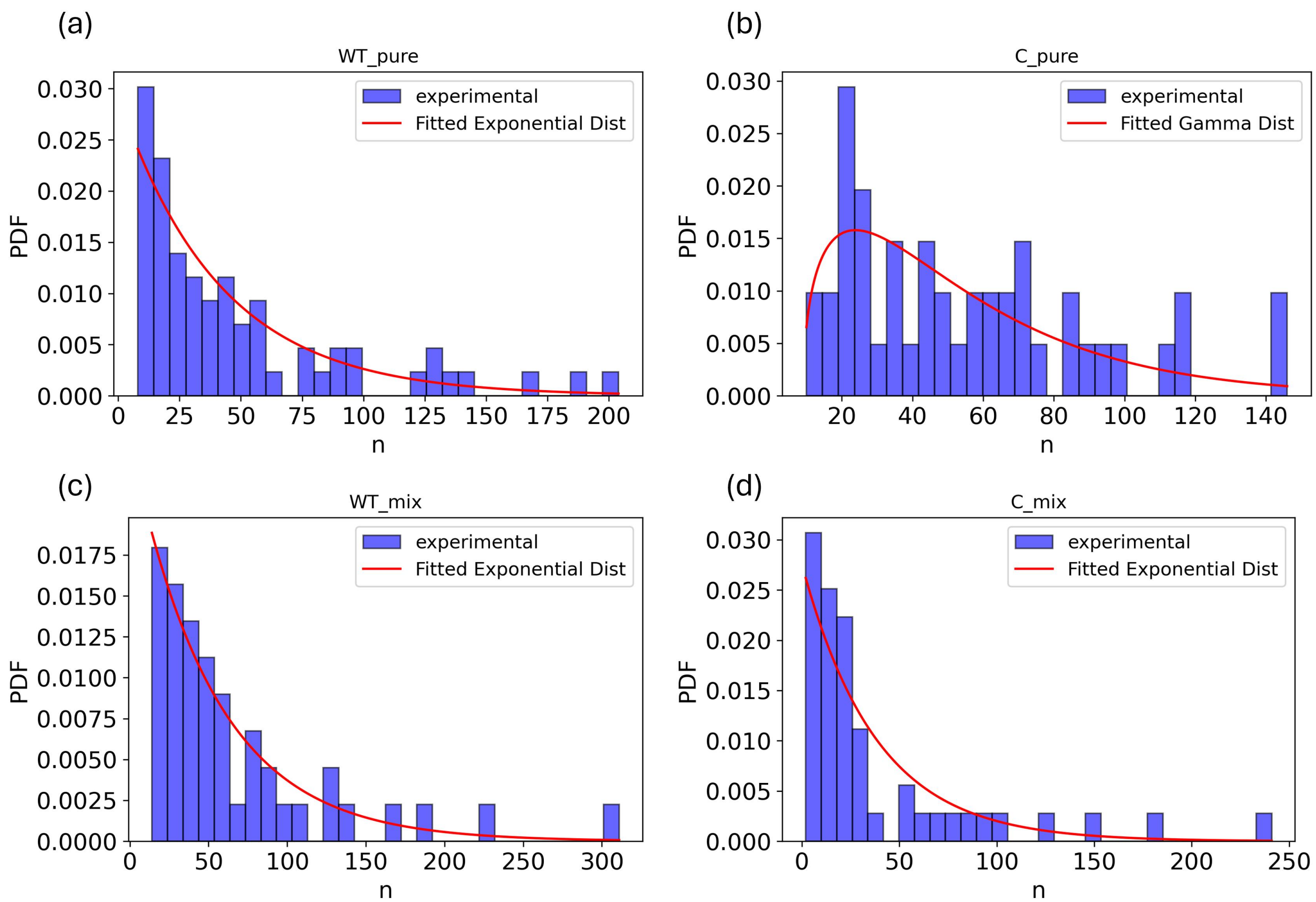

Fig. S1. The experimental data for the initial number of cells in organoids, as well as the fitted distributions. The panels show the data for (a) pure wild-type cells, (b) pure cancer cells, (c) wild-type cells in mixed conditions and (d) cancer cells in mixed conditions. The experimental data was taken from Ref. 1. The red curves indicate the fitted distribution to each set of data.

Table S1. Parameters of the fitted distributions to the initial number of cells in pure and mixed organoids. The parameters α, θ and n_0 are the shape, scale, and shift parameters, respectively.

	α	n_0	θ
pure wild-type	-	8.0	41.47
pure cancer	1.47	9.12	31.37
mixed wild-type	-	14.0	53.04
mixed cancer	-	2.0	38.18

Cost function

As mentioned in the main text, the cost function comprises three terms. The first term is related to the populations of wild-type and cancer cells in mixed organoids, the second term is for reproduction of the statistics of wild-type cells in different phases, and the third term for the effect of initial composition of organoids on the growth of each population. The total cost is calculated as in Eq. 62, which is the same as Eq. 28 in the manuscript.

$$L(\tilde{S}, \tilde{v}_{\mathrm{S}}) = \frac{1}{3}L_{\mathrm{pop}}(\tilde{S}, \tilde{v}_{\mathrm{S}}) + \frac{1}{3}L_{\mathrm{stat}}(\tilde{S}, \tilde{v}_{\mathrm{S}}) + \frac{1}{3}L_{\mathrm{init}}(\tilde{S}, \tilde{v}_{\mathrm{S}}). \quad [62]$$

The first term, L_{pop} is calculated using $W^*(t) = W(t)/W(0)$ and $C^*(t) = C(t)/C(0)$, the normalized populations of wild-type and cancer cells. Since the baseline for the population dynamics follows exponential growth, it is more appropriate to use the logarithms of $W^*(t)$ and $C^*(t)$ in the definition of the error. Thus, we have:

$$L_{\mathrm{pop}} = \frac{1}{2}\sum_i \omega_i^{\mathrm{W}} \left[\ln\left(W^*_{\mathrm{model}}(t_i)\right) - \ln\left(W^*_{\mathrm{exp}}(t_i)\right)\right]^2 + \frac{1}{2}\sum_i \omega_i^{\mathrm{C}} \left[\ln\left(C^*_{\mathrm{model}}(t_i)\right) - \ln\left(C^*_{\mathrm{exp}}(t_i)\right)\right]^2 \quad [63]$$

where i counts on the experimental data points, and the weight factors, ω_i^{W} and ω_i^{C} are calculated using the uncertainties for the experimental data, $\sigma_{\mathrm{exp}}^{\mathrm{W}}$ and $\sigma_{\mathrm{exp}}^{\mathrm{C}}$:

$$\omega_i^{\mathrm{W}} = \frac{1}{|\sigma_{\mathrm{exp}}^{\mathrm{W}}(t_i)/W^*_{\mathrm{exp}}(t_i)|^2}\left[\sum_j \frac{1}{|\sigma_{\mathrm{exp}}^{\mathrm{W}}(t_j)/W^*_{\mathrm{exp}}(t_j)|^2}\right]^{-1}, \quad [64]$$

and

$$\omega_i^{\mathrm{C}} = \frac{1}{|\sigma_{\mathrm{exp}}^{\mathrm{C}}(t_i)/C^*_{\mathrm{exp}}(t_i)|^2}\left[\sum_j \frac{1}{|\sigma_{\mathrm{exp}}^{\mathrm{C}}(t_j)/C^*_{\mathrm{exp}}(t_j)|^2}\right]^{-1}. \quad [65]$$

In order to make different terms of cost function to have the same order of magnitude in their numerical values, we need to multiply error terms by factors that are calculated based on typical scales of each of each cost terms. For the scale of the first term, we consider:

$$L^0_{\mathrm{pop}} = \frac{1}{2}\left\langle\left(\frac{\sigma_{\mathrm{exp}}^{\mathrm{W}}(t_i)}{W^*_{\mathrm{exp}}(t_i)}\right)^2\right\rangle_i + \frac{1}{2}\left\langle\left(\frac{\sigma_{\mathrm{exp}}^{\mathrm{C}}(t_i)}{C^*_{\mathrm{exp}}(t_i)}\right)^2\right\rangle_i = 4.9\times 10^{-3}, \quad [66]$$

where the averaging is done over the experimental data points.

For the second term, the error of statistics of wild-type cells in different phases, point out several requirements. Ideally, we want to fix the percentages of wild-type in three different states, for pure and mixed organoids. This means 6 quantities must be fixed. Since for each case (either pure or mixed), the fractions must sum up to 1.0, we need to fix 4 independent quantities. However, the fraction of differentiated wild-type cells (f_{D}) in pure conditions is already fixed in the model by Eq. 29. Hence, we need to fix three quantities. We choose the first one to be fraction of wild-type cells in S/G2/M phases in pure organoids. The second quantity is the ratio of the fractions of wild-type cells in mixed and pure organoids, $R_{\mathrm{S}} = \frac{f_{\mathrm{S,W,mix}}}{f_{\mathrm{S,W,pure}}}$. The third one is the same ratio, but for wild-type cells in G0 phase, i.e., $R_{\mathrm{G0}} = \frac{f_{\mathrm{G0,W,mix}}}{f_{\mathrm{G0,W,pure}}}$. From the experimental data, the target values for these ratios are $R^{\mathrm{tar}}_{\mathrm{S}} = 0.5(\frac{30}{47} + \frac{10}{20}) = 0.57$ and $R^{\mathrm{tar}}_{\mathrm{G0}} = \frac{29}{20} = 1.45$. We have used the experimental data presented in Fig. 4 in the manuscript to calculate these target values. Consequently, we consider the cost function of the statistics as follows

$$L_{\mathrm{stat}} = \left(\frac{1}{3}L_{\mathrm{S,pure}} + \frac{1}{3}L_{\mathrm{S,mix}} + \frac{1}{3}L_{\mathrm{G0,mix}}\right)\frac{L^0_{\mathrm{pop}}}{L^0_{\mathrm{stat}}}, \quad [67]$$

where

$$L_{\mathrm{S,pure}} = \left(f^{\mathrm{model}}_{\mathrm{S,W,pure}} - f^{\mathrm{exp}}_{\mathrm{S,W,pure}}\right)^2; \quad [68]$$

$$L_{\mathrm{S,mix}} = \left(f^{\mathrm{model}}_{\mathrm{S,W,pure}} - R^{\mathrm{tar}}_{\mathrm{S}}\, f^{\mathrm{model}}_{\mathrm{S,W,pure}}\right)^2; \quad [69]$$

$$L_{\mathrm{G0,mix}} = \left(f^{\mathrm{model}}_{\mathrm{G0,W,pure}} - R^{\mathrm{tar}}_{\mathrm{G0}}\, f^{\mathrm{model}}_{\mathrm{G0,W,pure}}\right)^2. \quad [70]$$

The factor $L^0_{\mathrm{pop}}/L^0_{\mathrm{stat}}$ makes this error term numerically comparable to the first term L_{pop}. According to the experimental data, the uncertainty for the fractions of wild-type cells in the G0 and S/G2/M phases is approximately $2.0 \sim 3.0\%$. So, we consider the scale of this term as

$$L^0_{\mathrm{stat}} = 0.03^2 = 9.0\times 10^{-4}. \quad [71]$$

For the last term of cost function, L_{init}, we take the following path. First, we fit lines to the logarithm of experimental growth of each type at $t = 60$ h –$\ln(W^*_{\mathrm{exp}}(60))$ or $\ln(C^*_{\mathrm{exp}}(60))$– as a function of the initial percentage of each cell type, i.e., $F_{\mathrm{W}}(0)$ and $F_{\mathrm{C}}(0)$ for wild-type and cancer cells, respectively. The data are the same as shown in Fig. 3 in the manuscript. We assumed that for wild-type cells the fitted line is $y = A + Bx$ and for cancer cells it is $y = D + Ex$. Least squares fitting provided

us with the coefficients $A = 0.749$, $B = 0.570$, $D = 3.362$ and $E = -1.966$. This allows us to write the third cost-function term as:

$$L_{\mathrm{init}} = \frac{1}{2}\frac{L^{0}_{\mathrm{pop}}}{L^{\mathrm{W},0}_{\mathrm{init}}} L^{\mathrm{W}}_{\mathrm{init}} + \frac{1}{2}\frac{L^{0}_{\mathrm{pop}}}{L^{\mathrm{C},0}_{\mathrm{init}}} L^{\mathrm{C}}_{\mathrm{init}}, \qquad [72]$$

where the error terms are

$$L^{\mathrm{W}}_{\mathrm{init}} = \left\langle [\, \ln\left(W^{*}_{\mathrm{model}}(60)\right) - (A + BF_{\mathrm{W}}(0))\,]^{2} \right\rangle_{\mathrm{model}}, \qquad [73]$$

and

$$L^{\mathrm{C}}_{\mathrm{init}} = \left\langle [\, \ln\left(C^{*}_{\mathrm{model}}(60)\right) - (D + EF_{\mathrm{C}}(0))\,]^{2} \right\rangle_{\mathrm{model}}. \qquad [74]$$

Again, the factors $L^{0}_{\mathrm{pop}}/L^{\mathrm{W},0}_{\mathrm{init}}$ and $L^{0}_{\mathrm{pop}}/L^{\mathrm{C},0}_{\mathrm{init}}$ rescale the error terms to be numerically comparable to L_{pop}, and the scales $L^{\mathrm{W},0}_{\mathrm{init}}$ and $L^{\mathrm{C},0}_{\mathrm{init}}$ are calculated as

$$L^{\mathrm{W},0}_{\mathrm{init}} = \mathrm{Var}\left[\ln\left(W^{*}_{\exp}(60)\right) - (A + BF_{\mathrm{W}}(0))\right]_{\exp} = 0.094; \qquad [75]$$

$$L^{\mathrm{C},0}_{\mathrm{init}} = \mathrm{Var}\left[\ln\left(C^{*}_{\exp}(60)\right) - (D + EF_{\mathrm{C}}(0))\right]_{\exp} = 0.26. \qquad [76]$$

Temporal variations of phases

As a complementary analysis, we present the model's predictions for the temporal evolution of phase-specific cell fractions in Fig. S2. In pure wild-type and cancer organoids, these fractions remain constant over time—a natural consequence of the quasi-steady-state assumption for the normalized cell density distribution. In mixed organoids, the growth factor concentration lies between the equilibrium values of pure wild-type and pure cancer cells. According to Eq. 1 in the manuscript, this results in a lower G1 progression speed for wild-type cells and a higher speed for cancer cells, compared to their respective pure cases. These differences lead to a reduction in the G1-to-S flux for wild-type cells, and an increase for cancer cells. Given that the S/G2/M progression speed v_{S} is assumed to be constant, wild-type cells accumulate in the G1 phase, while cancer cells become depleted. Consequently, the opposite occurs in the S/G2/M phases. In addition, due to the increased G1 fraction in wild-type cells, the fraction in G0 increases monotonically, as described by Eq. 6 in the main text. Moreover, these dynamics give rise to temporal fluctuations in the phase fractions, as the accumulation and depletion waves induced by mixing, propagate through the cell cycle and repeat periodically. However, these fluctuations gradually diminish as the total population grows, and the concentration asymptotically approaches the equilibrium value of cancer cells. Experimental data on the fractions of cancer cells, as well as the temporal variations for both types, are still lacking. Future measurements in this direction would allow for a more rigorous evaluation of the assumptions and predictions of the model.

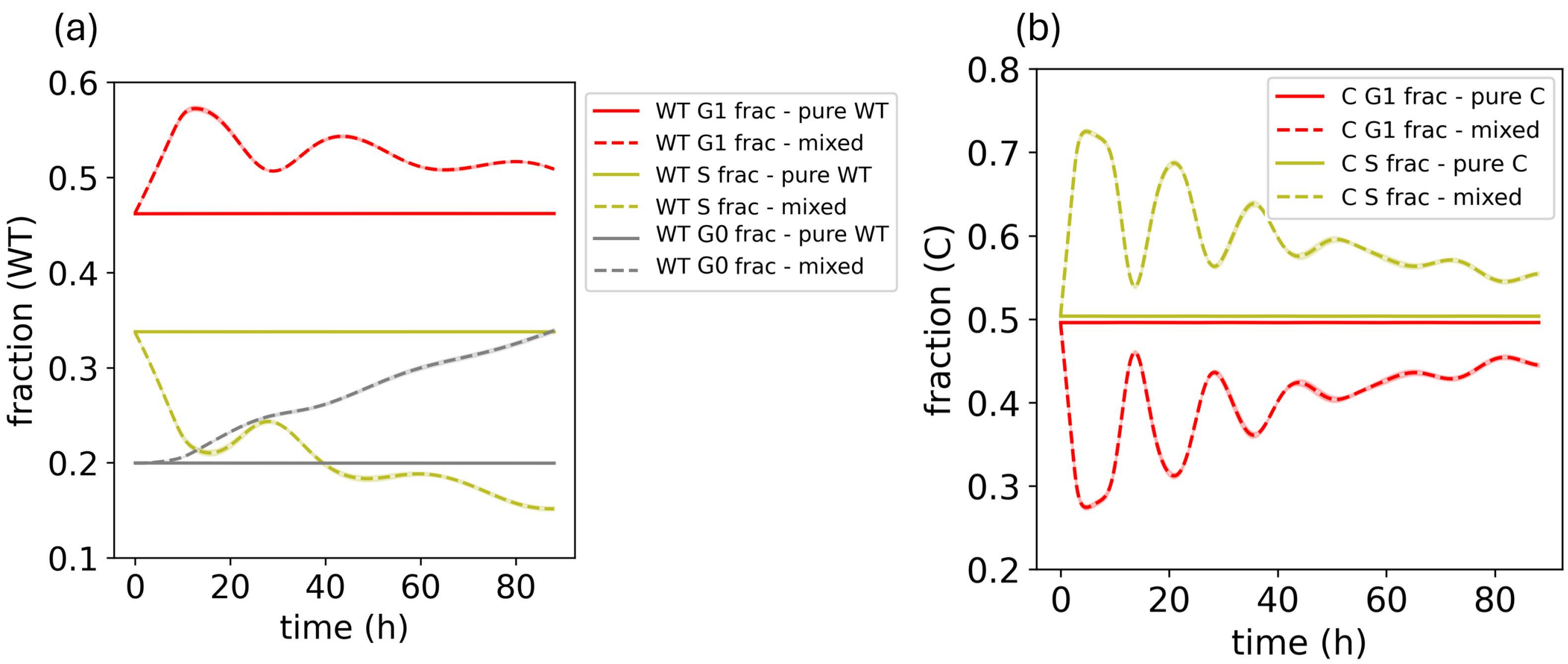

Fig. S2. Evolution of cell-cycle phase fractions over time. (a) Fractions of wild-type cells in the G1, S/G2/M, and G0 (differentiated) phases as a function of time. (b) Fractions of cancer cells in the G1 and S/G2/M phases over time. In both panels, solid lines represent pure organoids, while dashed lines indicate mixed conditions. Colors denote specific phases: red for G1, yellow for S/G2/M, and gray for G0 state. Note that cancer cells do not enter the differentiated phase.

Identifiability of the model

In this section, we study the identifiability of the model. The presented model has a number of fundamental parameters: $\tilde{S}$, $\tilde{v}_{\mathrm{S}}$, $\tilde{\mu}_{\mathrm{W}}$, $\tilde{\mu}_{\mathrm{C}}$, and $\tilde{r}_{\mathrm{D}}$. There are also a number of mediating parameters that are found using these fundamental ones, including $\tilde{\alpha}_{\mathrm{W}}$, $\tilde{\alpha}_{\mathrm{C}}$, m, and q — here we use the notation of Eqs. 1 - 59. To prove the local structural identifiability of the model, we need to show that for any arbitrary set of experimental data in the neighborhood of the actual data, all the parameters of model can be found uniquely.

The analytical derivations associated with Eqs. 1 - 59 show that once the information of pure conditions is known (f_{D} and γ), there are two independent parameters that must be fitted. The remainder can be found using this information. Without losing generality, we chose $\tilde{v}_{\mathrm{S}}$ and $\tilde{S}$ as the two independent parameters. The flowchart in Fig. S3(a) shows how the remaining parameters follow. The value f_{D} is an input from pure conditions, as is $\gamma = \beta_{\mathrm{C}}/\beta_{\mathrm{W}}$. To determine $\tilde{v}_{\mathrm{S}}$ and $\tilde{S}$, we scanned a range of values for these parameters and minimized the cost function $L(\tilde{S}, \tilde{v}_{\mathrm{S}})$ (introduced previously) over the corresponding two-dimensional parameter space. To demonstrate the local structural identifiability of the model, we show that, for a set of experimental data of the asymmetric population deviations, $\tilde{v}_{\mathrm{S}}$ and $\tilde{S}$ are found uniquely. Here, we take numerical approach to realize this as follows. The quantities $\overline{W}_{\mathrm{m}}/\overline{W}_{\mathrm{p}}$ and $\overline{C}_{\mathrm{m}}/\overline{C}_{\mathrm{p}}$ at time $t = 60$ h are taken as representative of the level of competition in mixed conditions and are shown in Fig. S3(b) and Fig. S3(c) as a function of $\tilde{S}$ and $\tilde{v}_{\mathrm{S}}$, respectively. Higher and the lower values of $\overline{C}_{\mathrm{m}}/\overline{C}_{\mathrm{p}}$ and $\overline{W}_{\mathrm{m}}/\overline{W}_{\mathrm{p}}$, respectively, are correlated with increased competition. Together the two define a complete description of the competition regime, as the former determines how much cancer cells benefit from the interaction, while the latter determines how much the wild-type cells are disadvantaged. It is clear that competition is strongest in the bottom-left corner of the diagrams and weakest in the top-right corner. Figure S3(d) shows both sets of contour lines together, which reveals that for any arbitrary regime of competition, represented by a pair of ratios $\overline{W}_{\mathrm{m}}(60\ \mathrm{h})/\overline{W}_{\mathrm{p}}(60\ \mathrm{h})$ and $\overline{C}_{\mathrm{m}}(60\ \mathrm{h})/\overline{C}_{\mathrm{p}}(60\ \mathrm{h})$, the parameters $\tilde{S}$ and $\tilde{v}_{\mathrm{S}}$ are uniquely found —at least in the neighborhood of the actual experimental values. The specific value can be obtained by intersecting the relevant contour lines, with the remaining parameters following uniquely from the flowchart in Fig. S3(a); assuming f_{D} and γ are given as part of the experimental data on pure conditions.

In addition to studying identifiability for the experimental values $f_{\mathrm{D}} = 0.2$ and $\gamma = 1.4$, we performed our analysis for several additional values of these parameters around this point to demonstrate robustness. We plot in Fig. S4 contour lines similar to those of Fig. S3(d), for $f_{\mathrm{D}} = 0.15$ and 0.25 (with $\gamma = 1.4$), and for $\gamma = 1.3$ and 1.5 (with $f_{\mathrm{D}} = 0.2$). The plots reveal that also for values of f_{D} and γ that depart from those observed, the two parameters $\tilde{S}$ and $\tilde{v}_{\mathrm{S}}$ are determined uniquely. Recall that the other parameters follow directly from this pair accordingly using the flowchart in Fig. S3(a).

In summary, combining analytical derivations and numerical analysis, we have shown (local) identifiability. That is, given an experimental data set that should be reproduced as the *output* of our model, all the internal parameters of the model are found uniquely. In the next section of SI, we will present a sensitivity analysis of the fitting cost function $L(\tilde{S}, \tilde{v}_{\mathrm{S}})$ on the values of the independent parameters $\tilde{S}$ and $\tilde{v}_{\mathrm{S}}$, keeping in mind that the uncertainties in the experimental data are encoded in the definition of the different terms in L (see Eqs. 62 - 76). As we will see, $\tilde{S}$ and $\tilde{v}_{\mathrm{S}}$ are found for the present set of experimental data with 95% confidence such that the uncertainty of each one does not go beyond 4% of the fitted value. This demonstrates that the model is also practically identifiable.

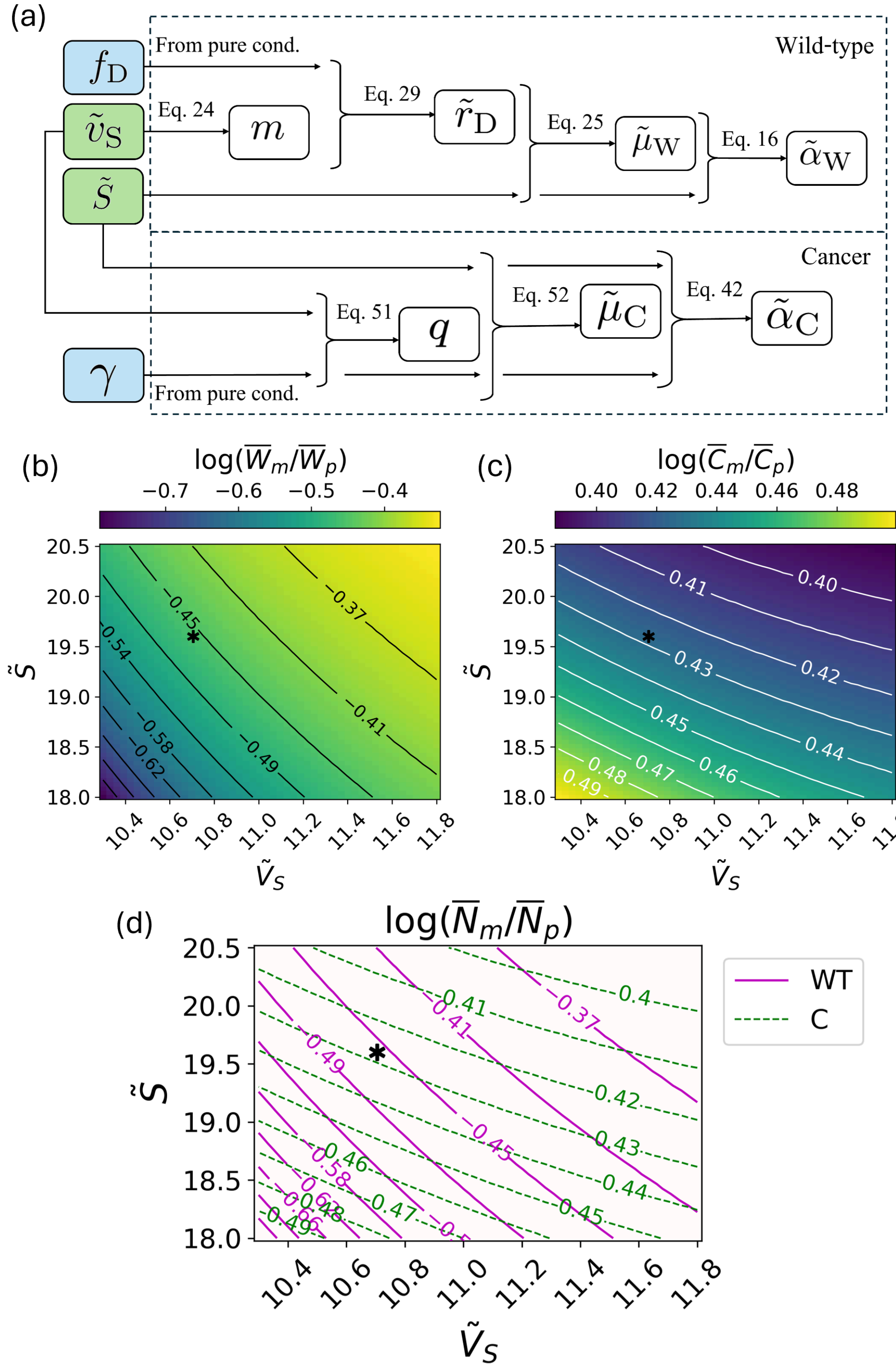

Fig. S3. Structural identifiability of the model. (a) Flowchart illustrating the procedure for determining the dependent parameters of the model. The independent parameters $\tilde{v}_{\mathrm{S}}$ and $\tilde{S}$ shown in green, are obtained through a fitting procedure. The differentiated fraction for pure wild-type cells, f_{D}, and the pure growth rate ratio $\gamma = \beta_{\mathrm{C}}/\beta_{\mathrm{W}}$ shown in blue, are inputs that follow from the experimental data in pure conditions. All remaining parameters for both types are subsequently computed using the specified equations. The numbering of the equations is internal to this supplement, *i.e.*, they do not refer to the main text. For additional clarity, we have separated the hierarchical dependence for wild-type and cancer cells using dashed-line boxes. (b) The effect of competition on wild-type cells, represented by $\log[\overline{W}_{\mathrm{m}}(60\ \mathrm{h})/\overline{W}_{\mathrm{p}}(60\ \mathrm{h})]$ as a function of $\tilde{S}$ and $\tilde{v}_{\mathrm{S}}$. (c) The effect of competition on cancer cells, represented by $\log[\overline{C}_{\mathrm{m}}(60\ \mathrm{h})/\overline{C}_{\mathrm{p}}(60\ \mathrm{h})]$ as a function of $\tilde{S}$ and $\tilde{v}_{\mathrm{S}}$. (d) Combined representation of the contour lines for $\log[\overline{N}_{\mathrm{m}}(60\ \mathrm{h})/\overline{N}_{\mathrm{p}}(60\ \mathrm{h})]$ from panels (b) and (c) as a function of $\tilde{S}$ and $\tilde{v}_{\mathrm{S}}$. The symbol N should be replaced by either W or C depending on the contour line. In panels (b)–(d), the subscripts m and p refer to mixed and pure conditions, respectively. The symbol $*$ indicates the fitted values of the parameters corresponding to the experiments, as explained in the main text.

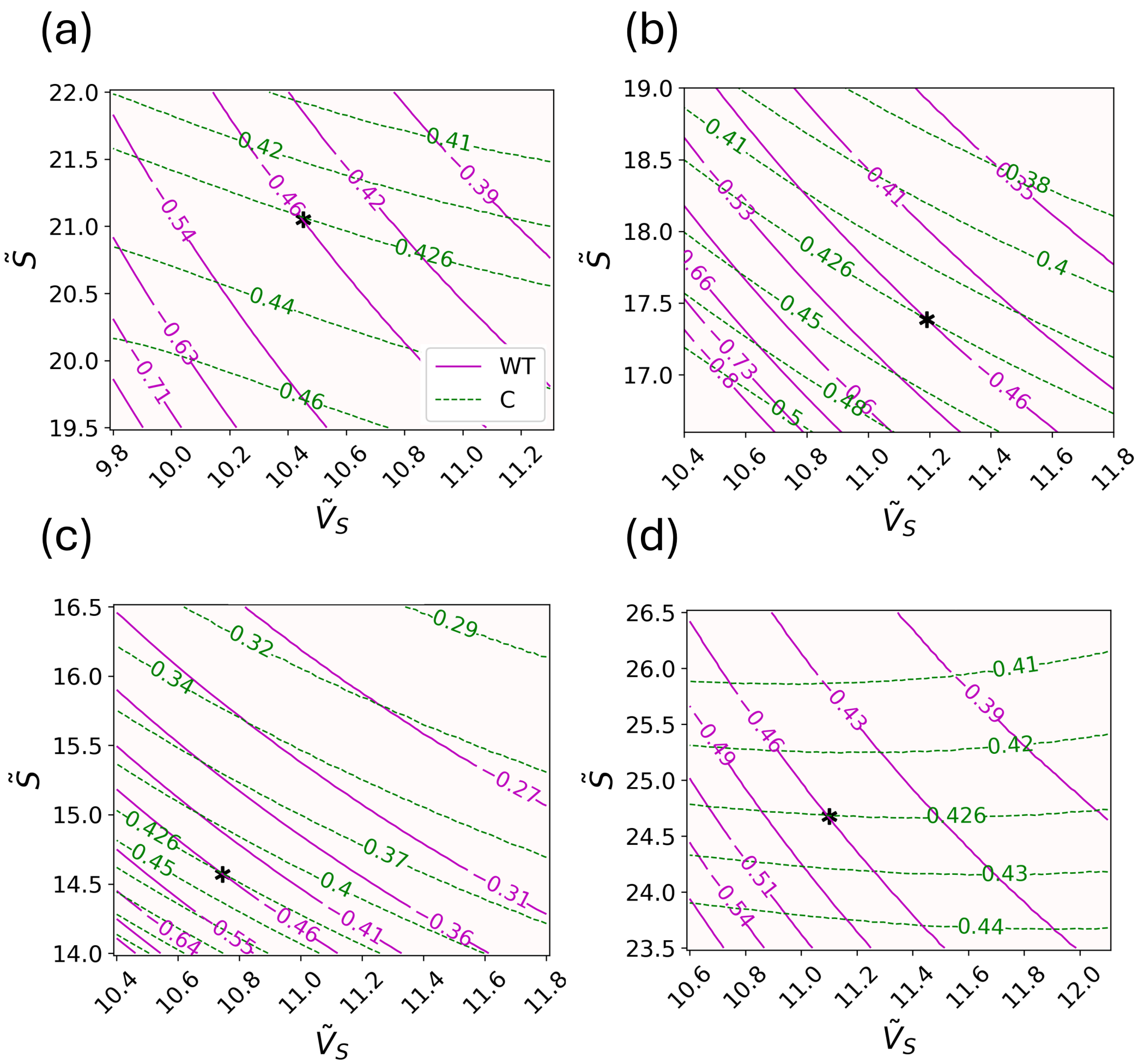

Fig. S4. Combined contour plots of $\log[\overline{N}_{\mathrm{m}}(60\ \mathrm{h})/\overline{N}_{\mathrm{p}}(60\ \mathrm{h})]$ as a function of independent fitting parameters $\tilde{S}$ and $\tilde{v}_{\mathrm{S}}$, for several values of pure-condition parameters f_{D} and $\gamma = \beta_{\mathrm{C}}/\beta_{\mathrm{W}}$. Here, N denotes either W or C, depending on the contour line, while the subscripts m and p refer to mixed and pure conditions, respectively. Panels (a) and (b) correspond to $f_{\mathrm{D}} = 0.15$ and $f_{\mathrm{D}} = 0.25$, respectively, both at $\gamma = \gamma^{\mathrm{exp}} = 1.4$. Panels (c) and (d) correspond to $\gamma = 1.3$ and $\gamma = 1.5$, respectively, both at $f_{\mathrm{D}} = f_{\mathrm{D}}^{\mathrm{exp}} = 0.2$. The superscript "exp" denotes parameter values obtained from the present experimental data. The legend shown in panel (a) applies to all panels. In all panels, the symbols $*$ indicate points corresponding to the same values of $\overline{W}\mathrm{m}(60\ \mathrm{h})/\overline{W}\mathrm{p}(60\ \mathrm{h})$ and $\overline{C}\mathrm{m}(60\ \mathrm{h})/\overline{C}\mathrm{p}(60\ \mathrm{h})$ as the identically marked point in Fig. S3(d).

Sensitivity analysis

In this section, we present an analysis on sensitivity of the model to the independent parameters. We first verified that we had minimized the cost function 62 by studying its dependence on the parameters $\tilde{v}_\mathrm{S}$ and $\tilde{S}$. Specifically, we varied $\tilde{v}_\mathrm{S}$ from 10.3 to 11.8 and $\tilde{S}$ from 18.5 to 20.5, using step sizes of $d\tilde{v}_\mathrm{S} = 0.0306$ and $d\tilde{S} = 0.0424$, respectively. These intervals cover at least $\pm 4.6\%$ of $\tilde{v}_\mathrm{S}$, and $\pm 4.7\%$ of $\tilde{S}$ at the point of the minimum cost. For each parameter pair, we computed the average cost according to Eq. 62 over all the *in-silico* mixed organoids. A total of 700 random initial cell sizes were generated per parameter pair, using the fitted distributions for initial sizes. The average cost was then calculated across the full set of samples, as well as within 14 equal-sized subsets (blocks) of 50 samples each. The full set of data points was used to identify the location of the global minimum, while the subsets served to generate independent samples of that location. Figure S5 shows the heatmap of the calculated cost function, along with the location of the minimum and its independent samples. To perform a sensitivity analysis of the cost function, we evaluated the Hessian matrix of it by fitting a weighted paraboloid of the form $L \approx a_1 \tilde{v}_\mathrm{S}^2 + a_2 \tilde{S}^2 + a_3 \tilde{v}_\mathrm{S} \tilde{S} + a_4 \tilde{v}_\mathrm{S} + a_5 \tilde{S} + a_6$ to a 9×9 grid of data points centered around the identified global minimum. The second-order partial derivatives obtained from this fit define the entries of the Hessian matrix $\mathbf{H}_L$, which reads:

$$\mathbf{H}_L = \begin{bmatrix} \dfrac{\partial^2 L}{\partial \tilde{v}_\mathrm{S}^2} & \dfrac{\partial^2 L}{\partial \tilde{v}_\mathrm{S}\, \partial \tilde{S}} \\ \dfrac{\partial^2 L}{\partial \tilde{S}\, \partial \tilde{v}_\mathrm{S}} & \dfrac{\partial^2 L}{\partial \tilde{S}^2} \end{bmatrix} = \begin{bmatrix} 2a_1 & a_3 \\ a_3 & 2a_2 \end{bmatrix} = \begin{bmatrix} 0.0104 & 0.0034 \\ 0.0034 & 0.0014 \end{bmatrix}. \qquad [77]$$

Since the determinant and the trace of $\mathbf{H}_L$ are both positive, the captured critical point is a minimum for the cost function. Moreover, the eigenvectors of the Hessian matrix define the directions for the highest and the lowest increase in the cost function when deviating from the minimum. For the $\mathbf{H}_L$ above, the eigenvalues and the eigenvectors are $\lambda_1 = 1.15 \times 10^{-2}$ and $\lambda_2 = 2.75 \times 10^{-4}$, with eigenvectors $\mathbf{v}_1 = [0.95, 0.31]$ and $\mathbf{v}_2 = [-0.31, 0.95]$, the first and the second components being in the direction of $\tilde{v}_\mathrm{S}$ and $\tilde{S}$, respectively. The directions for the steepest and the lowest variations are shown in Fig. S5 with red and blue dashed lines.

Moreover, we used two different methods to determine uncertainty in localization of the minimum-cost point. First, we identified the region in which the cost function varies by less than 5% from its minimum value. This region is shown by the white dashed line in Fig. S5. As evident in the figure, the region forms an elongated ellipse tilted along the direction of lowest curvature of the cost function. This manifests as anisotropy in the sensitivity of the cost function with respect to variations in $\tilde{v}_\mathrm{S}$ and $\tilde{S}$. Within this region, each parameter changes by at most approximately 4% from its fitted value. As a second approach, we used independent samples of the minimum-cost location to estimate a 95% confidence region. Assuming that the variability in the sampled locations arises from a bivariate normal distribution, this confidence region is represented by an ellipse centered at the mean of the samples. Its shape and orientation are determined by the covariance matrix of the samples. The boundary of the ellipse corresponds to points whose squared Mahalanobis distance from the mean equals the 95th percentile of the chi-square distribution with two degrees of freedom, which is $\chi_2^2 \approx 5.99$. This region is shown with a solid white line in Fig. S5.

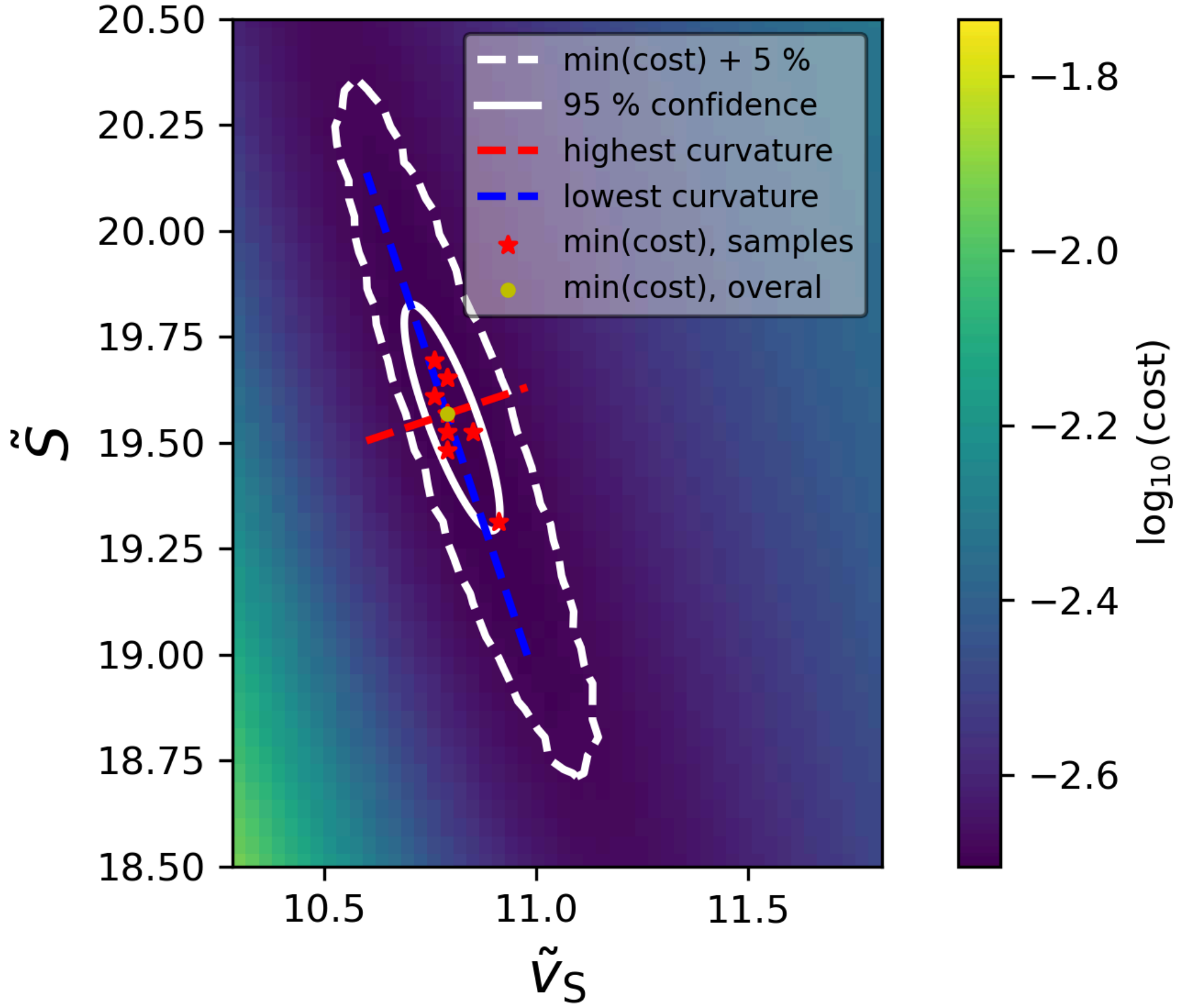

Fig. S5. Dependence of the cost function in Eq. 62 on the independent parameters $\tilde{v}_S$ and $\tilde{S}$. The heatmap shows $\log_{10}(L(\tilde{S}, \tilde{v}_S))$ as a function of these parameters, with the color scale indicated by the color bar. The step sizes for $\tilde{v}_S$ and $\tilde{S}$ are 0.0306 and 0.0424, respectively. For each parameter pair, 400 initial cell numbers were independently drawn from the synthetic initial size distributions for mixed organoids, and the average cost was computed. The yellow dot marks the location of the global minimum cost. The white dashed contour line indicates the region where the cost increases by 5% relative to the minimum. Red and blue dashed lines represent the directions of highest and lowest curvature in the cost landscape, respectively. Red stars denote independently estimated minimum-cost locations, obtained from subsets (blocks) of the data. The solid ellipse shows the 95% confidence region for the estimated position of the global minimum.

Dependence of competition on the differentiation rate

To show the influence of the uptake capacity ratio $r_\mu = \tilde{\mu}_{\mathrm{C}}/\tilde{\mu}_{\mathrm{W}}$, on the intensity of competition, we studied how the normalized populations in mixed conditions deviate from those in pure conditions as the ratio r_μ is varied. This is shown in Fig. 5 of the main text, which we will reference throughout this section. The data presented in Fig. 5(a) shows that even for $r_\mu = 1$, there is a difference between pure wild-type and cancer population growth. This difference is caused by differentiation, which is present for wild-type cells and absent for cancer cells.

In this section, we study the effect of the ratio r_μ and the differentiation rate $\tilde{r}_{\mathrm{D}}$ simultaneously to evaluate their relative importance in the regulation of competition. We varied these quantities in the intervals $\tilde{r}_{\mathrm{D}}/\tilde{r}^*_{\mathrm{D}} \in [0.1, 8.0]$ and $r_\mu \in [1.0, 16.0]$, where $\tilde{r}^*_{\mathrm{D}}$ is the fitted value presented in the main text. We kept the quantities $\tilde{S}$, $\tilde{\mu}_{\mathrm{W}}$, $\tilde{v}_{\mathrm{S}}$ equal to the values presented in the main text, and varied r_μ by changing $\tilde{\mu}_{\mathrm{C}}$, along with the differentiation rate $\tilde{r}_{\mathrm{D}}$. Then we looked at the normalized populations of each type and the differentiated fraction of wild-type cells, again in analogy to Fig. 5. In line with the experiments, we chose the time $t = 60$ h to examine these quantities.

Figure S6 shows how the intensity of competition is regulated by r_μ and $\tilde{r}_{\mathrm{D}}$. Similar to Fig. 5(a), we first studied the pure conditions. Panels (a) and (b) of Fig. S6 show the average of normalized populations of pure wild-type and cancer cells at $t = 60$ h. As one expects, the population decreases for wild-type cells with increasing differentiation rate $\tilde{r}_{\mathrm{D}}$, independent of the relative uptake r_μ. Conversely, for cancer cells, the population increases when the relative uptake r_μ grows, which is independent of $\tilde{r}_{\mathrm{D}}$, as these cells do not differentiate.

In analogy to Fig. 5(b,c), we plotted the ratios of normalized populations, $\overline{W}_{\mathrm{m}}/\overline{W}_{\mathrm{p}}$ and $\overline{C}_{\mathrm{m}}/\overline{C}_{\mathrm{p}}$ as the primary indicators of competition intensity. Panels (c) and (d), respectively, of Fig. S6 show these quantities as a function of r_μ and $\tilde{r}_{\mathrm{D}}$. The graphs reveal that deviations from the pure conditions depend predominantly on the ratio r_μ, while they are only weakly dependent on the differentiation rate.

As a secondary measure of competition, we have plotted the fraction of the differentiated wild-type cells in panels (e) and (f) of Fig. S6 for pure and mixed condition, respectively. In pure conditions, the differentiated fraction is independent of r_μ, as expected, and increases with $\tilde{r}_{\mathrm{D}}$. For the mixed conditions, the ratio r_μ becomes influential, as cancer cells lead to an accumulation of wild-type cells in the G1 phase, where they might start differentiation. This is in line with the results shown in Fig. 5(d). However, as the the effect of r_μ on the differentiation is indirect, in contrast to the dependence on $\tilde{r}_{\mathrm{D}}$, it is plays a subdominant role.

SI Dataset S1 ()

The data used in this document are available in the data package referenced in the main manuscript.

References

1. A Krotenberg Garcia, et al., Cell competition promotes metastatic intestinal cancer through a multistage process. *iScience* **27**, 109718 (2024).

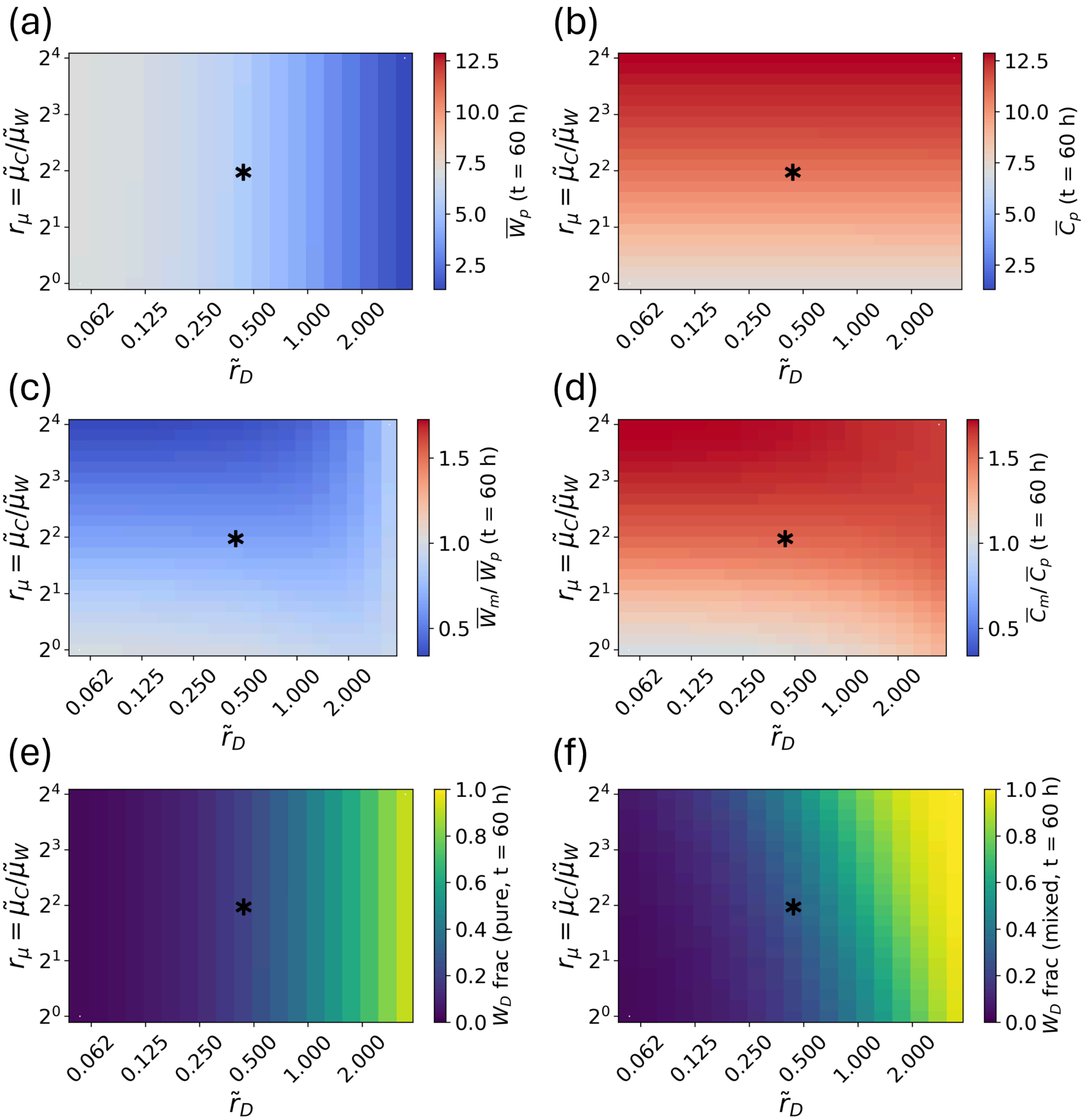

Fig. S6. Dependence of the competition on the uptake capacity ratio (r_μ) and the differentiation rate ($\tilde{r}_\mathrm{D}$). (a, b) The average normalized population of (a) wild-type and (b) cancer cells in pure conditions. (c, d) Ratios of the normalized populations in mixed and pure conditions for (c) wild-type and (d) cancer cells. (e, f) The fraction of differentiated wild-type cells in (e) pure and (f) mixed conditions. All the data refers to the time $t = 60$ h. The symbol $*$ indicates the fitted value for the quantities r_D and r_μ presented in the main text. All the panels have logarithmic scale with the base 2 on the x and y axes.
